# Hierarchical Coarse-Grained Strategy for Macromolecular Self-Assembly: Application to Hepatitis B Virus-Like Particles

**DOI:** 10.3390/ijms232314699

**Published:** 2022-11-24

**Authors:** Philipp Nicolas Depta, Maksym Dosta, Wolfgang Wenzel, Mariana Kozlowska, Stefan Heinrich

**Affiliations:** 1 Institute of Solids Process Engineering and Particle Technology (SPE), Hamburg University of Technology, 21073 Hamburg, Germany; 2 Boehringer Ingelheim Pharma GmbH & Co Kg., 88400 Biberach an der Riss, Germany; 3 Institute of Nanotechnology (INT), Karlsruhe Institute of Technology, 76344 Eggenstein-Leopoldshafen, Germany

**Keywords:** multiscale modeling, molecular discrete element method, supervised learning, macromolecular self-assembly, capsid formation, hepatitis B VLP

## Abstract

Macromolecular self-assembly is at the basis of many phenomena in material and life sciences that find diverse applications in technology. One example is the formation of virus-like particles (VLPs) that act as stable empty capsids used for drug delivery or vaccine fabrication. Similarly to the capsid of a virus, VLPs are protein assemblies, but their structural formation, stability, and properties are not fully understood, especially as a function of the protein modifications. In this work, we present a data-driven modeling approach for capturing macromolecular self-assembly on scales beyond traditional molecular dynamics (MD), while preserving the chemical specificity. Each macromolecule is abstracted as an anisotropic object and high-dimensional models are formulated to describe interactions between molecules and with the solvent. For this, data-driven protein–protein interaction potentials are derived using a Kriging-based strategy, built on high-throughput MD simulations. Semi-automatic supervised learning is employed in a high performance computing environment and the resulting specialized force-fields enable a significant speed-up to the micrometer and millisecond scale, while maintaining high intermolecular detail. The reported generic framework is applied for the first time to capture the formation of hepatitis B VLPs from the smallest building unit, i.e., the dimer of the core protein HBcAg. Assembly pathways and kinetics are analyzed and compared to the available experimental observations. We demonstrate that VLP self-assembly phenomena and dependencies are now possible to be simulated. The method developed can be used for the parameterization of other macromolecules, enabling a molecular understanding of processes impossible to be attained with other theoretical models.

## 1. Introduction

The specific function of a bioactive macromolecule is encoded in its chemical composition, three-dimensional (3D) structure, and self-assembly affinity. Even if the determination of the building units of macromolecules is nowadays a regular procedure in many laboratories worldwide, the latter properties are still cumbersome and not fully understood. This limitation originates often from the relatively weak non-covalent interactions: either within the macromolecule or between several macromolecules, that cannot be easily measured experimentally and calculated theoretically. Therefore, many of biologically relevant processes are constantly under investigation, hindering the successful treatment of harmful diseases such as cancer, viral infections, Alzheimer’s disease, etc., which are often based on misfolding of proteins and mistakes in their self-assembly and aggregation. On the one hand, unwanted changes are often caused genetically, i.e., by the subtle differences in the chemical state in the cell environment or via mutations, therefore, they are determined on a molecular scale. On the other hand, they happen on relatively long timescales that are not accessible by most of the first principle atomistic methods due to the high computational effort, which strongly limits investigated time and length scales.

Recent years are known by the prominent developments of various simulation methods to upscale calculations, including quantum mechanics/molecular mechanics (QM/MM) [1], accelerated molecular dynamics (MD) [2], replica-exchange MD [3,4], kinetic Monte Carlo [5], as well as multi-scale simulations accounting different coarse-graining (CG) of molecular systems [6,7,8,9], representation of solvent [10,11,12], and more [13,14]. In addition, the coupling of the micro- and macroscales has been shown to be realized in serial or in parallel [15] to perform macroscopic simulations including results from microscale models. The up-scaling of all-atom molecular dynamics (AA-MD) simulations to different types of CG, i.e., coarse-grained molecular dynamics (CG-MD) [6,9,16] is one of the most commonly used approaches. Here, groups of atoms are represented as larger single beads [17] allowing to significantly reduce degrees of freedom (DOF) and permitting an increase in the timestep, thus enabling simulations of large macromolecules for longer times. Various types of CG methods exist around Langevin dynamics (LD) [18,19], Brownian dynamics (BD) [20], and dissipative particle dynamics (DPD) [21]. In the same context, various coarse-grained force-fields (FF) for a range of systems have been developed, including Martini [22,23,24], SIRAH [25,26], UNRES [27], CABS [28], and others.

The application of methods mentioned allows to jump from the nanometer length and nanosecond time scales to the tens-hundreds nanometers and microseconds. However, the efficient construction of CG models is related to numerous challenges. The main complexity related to the development of up-scaling strategies is an appropriate parameterization of models on higher scales [17]. While various bottom-up strategies exist based on first-principles (e.g., thermodynamic integration [29], free energy perturbation [30], umbrella sampling [31]), these methods are often not sufficient leading either to fully empirical force-fields using a top-down approach or hybrid approaches (e.g., the Martini FF [23]). Similarly, the transferability decreases with increasing levels of coarse-graining. With regard to their formulation, the majority of such FF employ the same 1D neoclassical distance-based functional descriptions [32] as AA-MD, thus employing the same approximation as a point object for groups of atoms. Recently, machine learning (ML) methods in the context of, e.g., artificial neural networks (ANN) and Gaussian process regression (GPR), have gained increasing interest for both formulation and parameterization of CG models [33,34,35]. However, ML-based FF have largely focused on ANN and been developed/applied to small molecules or ordered solids [35]. Similarly, GPR methods have, to our knowledge, only been applied up to four DOF for the potential energy surface in the CG model and for small molecules such as methanol and benzene [36], as well as alanine tripeptide [37,38].

**Figure 1:**
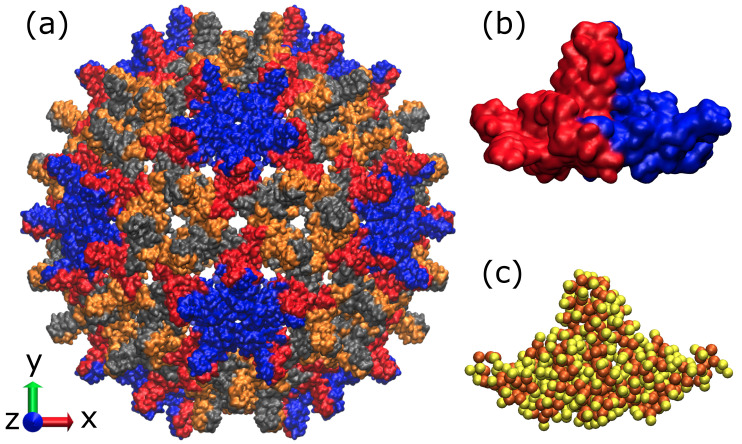
Atomistic reference structure of (**a**) HBcAg T = 4 capsid (composed of 120 dimers/240 monomers) and (**b**) HBcAg${}_{2}$ dimer based on PDB 6HTX [56] and PDB 1QGT [43]. (**c**) The coarse-grained Martini representation after representative clustering.

Even thought some of the methods described above permit efficient simulations of macromolecules including viruses [39,40,41,42], their application to the simulation of virus self-assembly, e.g., virus capsid formation, is limited. Such simulations require hundreds or thousands of large protein macromolecules that have to be simulated on the millisecond (or longer) timescale. One such example is the formation of virus-like particles (VLPs). Here, the hepatitis B virus (HBV) VLPs (see Figure 1a) [43] are the most studied assembly systems that are used in many vaccines [44,45] and drug delivery [46,47] systems nowadays. On contrary to HBV made out of core proteins (C${}_{p}$) with 183 amino acids (aa), VLPs can be self-assembled from C${}_{p}$ with 149 aa that were shown to be the main domain of the protein taking part in the capsid self-assembly (the rest of the 183 aa chain is binding viral DNA or RNA) [48,49]. Typically, two types of HBV VLPs of different icosahedral symmetry, i.e., of T = 3 and T = 4, are formed from 180 and 240 C${}_{p}$, respectively. Most of such VLPs (95%) were shown to be of T = 4 symmetry; however, it is strongly dependent on experimental conditions [50,51], which are modulating the strength of protein–protein interactions, thus capsid intermediates [52,53]. However, trapping these intermediates is nearly impossible, because the self-assembly is a nucleation-limited process [51,54]. Thus, the smallest intermediates captured experimentally (after pentamer of HBcAg dimers [50,55], see blue structures Figure 1a) are 104-108-mers, 110-111-mers and 117-mers [53]. The observation of capsid nucleus and intermediates obtained out of dimers of C${}_{p}$, i.e., HBcAg${}_{2}$ (denoting subsequently HBcAg dimer), that are known to be the smallest building unit of a capsid [49], was never done in silico on a large scale.

When going to larger scales beyond traditional coarse-graining, which are especially interesting for supramolecular assemblies such as VLPs, the challenge is two-fold. Firstly, models become increasingly specialized and less transferable. Thus, their creation becomes a trade-off between the cost of formulation/parameterization and the value of increased modeling scales, therefore making transferable approaches preferential. Secondly, at the ultra-coarse-grained level (specifically when abstracting entire macromolecules as a CG bead), capturing their orientation becomes crucial. This results in the interactions of such FF being six-dimensional (6D), making both the formulation and parameterization challenging. While some models exist in this context, specifically for molecular capsids, they are largely employing heavily simplified geometries, i.e., patchy-spheres, trapezoidal/triangular shapes, or hard pseudoatoms [57,58,59,60] and, consequently, are difficult to re-parameterize for different systems, as also to understand pathways of capsid self-assembly.

**Figure 2:**
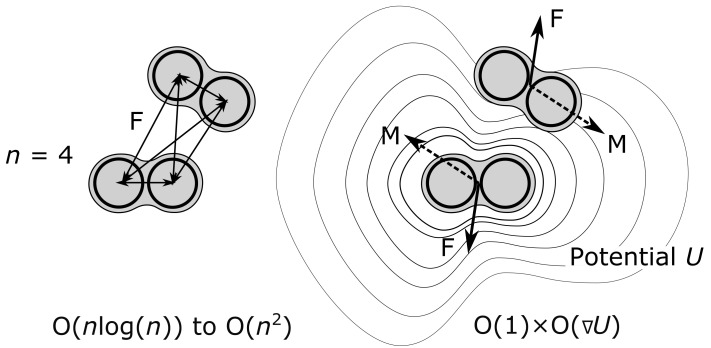
Effect of macromolecule abstraction as anisotropic beads with interaction potential on computational complexity (*n* is number of atoms, neglecting solvent and ions). Note that a single interaction of HBcAg${}_{2}$ is equivalent to n=9432 and further increased by the solvent atoms ($n\approx 10^{5}$).

In order to improve these aspects, we present the development of a multiscale model framework based on abstracting entire macromolecules as anisotropic beads. These beads possess a position, orientation, and spatial extent along with data-driven models derived from MD describing interaction with the environment and between the beads. We focus on deriving a generally applicable approach for intermolecular interaction potentials (*U*) between the beads through data-driven fields (6D), on which a gradient operation (−∇U) is carried out to determine forces and torques of pairwise contacts, see schematically in Figure 2. This approach reduces the complexity of a pairwise macromolecular contact with *n* atoms from between O(nlog(n)) and O($n^{2}$) 1D neoclassical distance-based atom contacts [32] to that of a single gradient operation on the intermolecular interaction potential O(∇U)-thus drastically reducing computational requirements, permitting increased time steps, and maintaining high levels of detail in the potential field. The desired 6D intermolecular interaction potentials are derived from MD using Kriging [61,62,63,64], which provided the best linear unbiased estimate (BLUE) of the potential in a ’white-box’ model fashion (i.e., available for inspection). In addition to intermolecular interaction, anisotropic diffusion and the respective thermodynamics of these abstracted molecules (resulting from the solvent environment) is modeled through an implicit Langevin dynamics approach using the the previously published method [65] along with MD parameterization. The developed multiscale methodology is applied to the self-assembly of hepatitis B VLPs starting from the dimers of core proteins up to capsids of icosahedral T = 3 and T = 4 symmetry. It is shown to capture the complexity of self-assembly including multiple assembly pathways, capsid-like intermediates, as well as assembly kinetics.

## 2. Methods and Materials

**Figure 3:**
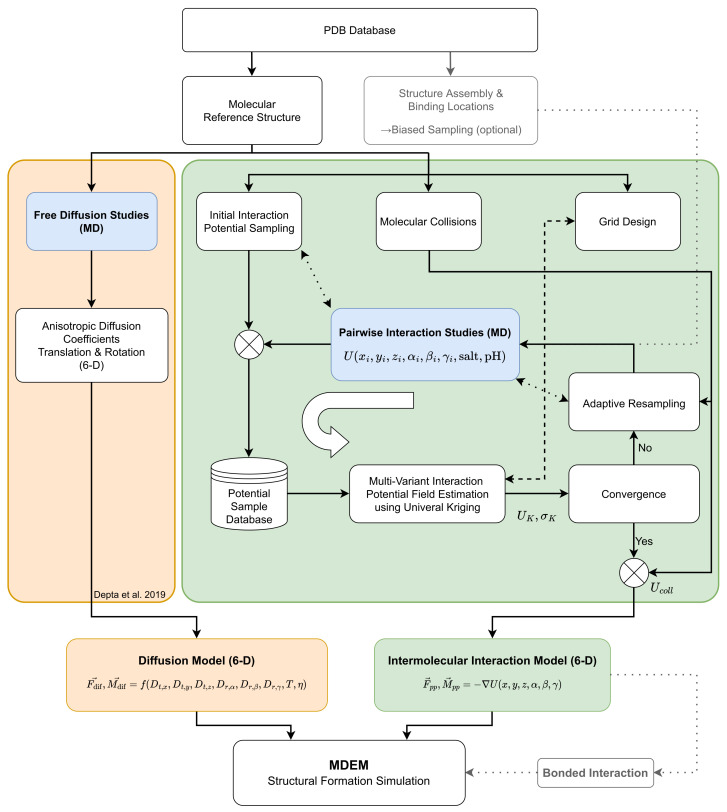
Framework overview with a focus on intermolecular interaction. For details on the diffusion model see ref. [65]. Blue indicates MD simulations, greyed-out regions are optional components, dotted lines indicated usage of related functionality (i.e., MD simulation is performed), dashed lines indicate information exchange.

As outlined in the introduction, to gain insight into the self-assembly of molecular systems, such as HBcAg, through non-covalent interactions we have developed a generically formulated data-driven framework for describing macromolecular interactions on the micrometer size and millisecond time scale. For this, each macromolecule is abstracted as an object with a position and orientation and all its anisotropic properties, e.g., the interaction with environment and other molecules (including spatial extend), were captured through data-driven models parameterized from CG-MD. This level of abstraction is termed by us as the Molecular Discrete Element Method (MDEM), indicating the intermediate level between MD and DEM. An overview of this multiscale model framework including the parameterization approach is schematically depicted in Figure 3 and explained in detail below. It is applied here to HBcAg${}_{2}$ proteins (see Figure 1), but can be transferred to any macromolecule or assembly process of interest using a semi-automated parameterization procedure.

### 2.1. Framework Overview

At the basis of the framework is the atomic reference structure of the macromolecule (e.g., from the Protein Data Bank, PDB), including optional information on structural assembly and binding locations. Two model components (denoted in light orange and green in Figure 3 with MD parameterization in blue) are then used to describe interactions of each abstracted molecule with the environment and other molecules, i.e., homodimeric proteins in the case of the VLPs. The first one (orange) is an anisotropic force-based diffusion model based on Langevin dynamics that is used to describe the interaction of a macromolecule with the (implicit) solvent environment and enforce the desired canonical ensemble. The second one (green) is an intermolecular interaction model, which describes the interaction between macromolecules. The latter consists of a data-driven interaction potential derived from MD simulations (blue) using a Kriging-based strategy, which is then used as a 6D potential field during the simulation through a numerical gradient operation to derive interaction forces and torques. Note that in this context, by ’conformation’ we refer to the structure of a molecule, and by ’configuration’ we refer to the relative position and orientation of a molecule B in the body frame of a molecule A, i.e., 6D interaction space with Cartesian coordinates x,y,z and Euler angles α,β,γ. The proposed framework accounts for the inclusion of processes parameters such as temperature, pH, salt, viscosity, some of which can be altered without requiring a re-parameterization, i.e., temperature and viscosity, as long as the reference structure is stable in the desired conditions. The framework implementation and scaling in the context of high-performance computing was published separately for reporting to the High-Performance Computing Center Stuttgart [66].

The main advantage of the proposed methodology is that mesoscales can be investigated, which are much larger in time and length than traditional CG-MD. Furthermore, due to the generic formulation of the models, the framework is flexible to be adapted to other systems. This is especially true concerning the intermolecular interaction model, which possesses significantly more freedom to describe interaction over, e.g., a functional description in 6D space. At the same time, assumptions and simplifications apply. The flexibility of the molecular reference structure is captured implicitly in the diffusion and interaction model, consequently implicitly considering internal degrees of freedom. Furthermore, the diffusion model is parameterized for dilute systems and the diffusion restriction during structural formation captured only implicitly through the interaction model. Hydrodynamic interaction is neglected in agreement with literature for the investigated anisotropic biomolecules [67]. The quality of parameterization depends on the underlying MD model for parameterization, which is elaborated in the results section. Further, in the chosen approach some thermodynamic accuracy is sacrificed in order to gain computational practicality for the 6D space. Lastly, due to the field formulation of the interaction model, limitations of resolution apply from memory constraints.

### 2.2. Reference Structure HBcAg${}_{2}$ Dimer

A visualization of the icosahedral capsid with T = 4 symmetry, determined by X-ray crystallography [43], is shown in Figure 1a. As introduced above, it consists of 120 HBcAg dimers, denoted as HBcAg${}_{2}$ (Figure 1b). They are of quasi-equivalent nature (known as AB and CD dimers) with slightly different conformations based on some disorder in the spike tips of CD, shorter gaps between its constituent chains, and differences in the interdimer interaction regions (particularly residues 128–136) [43]. As these regions are flexibly modeled along with the entire molecule in the MD parameterization of the proposed framework, only one reference structure was determined by representative clustering.

The atomistic reference structure of HBcAg${}_{2}$ was prepared using two structures of different resolution reported in the PDB, i.e., 6HTX [56] and 1QGT [43]. Residues 74 and 97 of mutated 6HTX were reverted to the wild type C${}_{p}$ residues and reconstructed by *ROSETTA 3.8* [68] using 1QGT as a template. The missing residues of the C-terminal chain were added by loop homology modeling using Modeller 9.21 [69]. The corresponding coarse-grained reference structure (see Figure 1c) was determined using representative clustering of on the *martinized* structure [22]. For representative clustering, the linkage method, as implemented in *Gromacs* version 5.1.1 [70], was applied to dimer conformations obtained from a 10 ns CG-MD run at 293 K and 150 mM NaCl. The root-mean-square (RMS) deviation of the determined representative structure with respect to the reference conformation was 0.39 nm. The structure was oriented along its principle component axes in descending order to provide the body reference frame at the center of mass. The radius of gyration of a dimer was measured as 1.31 nm, 1.85 nm and 1.97 nm in x,y,z, respectively.

### 2.3. Intermolecular Interaction Potential

In order to account for the interactions between macromolecules driving their self-assembly, we have developed a computational scheme aimed to derive intermolecular potentials from MD using Universal Kriging. In this section, we explain key components of this multiscale scheme, including the MD model, spatial descriptors used for describing configurations, basic functions for trend and variogram modeling, details on Universal Kriging for multivariant estimation, the field grid design, as well as a 2D example of the methodology and details of the implementation. The reported model has been applied for the VLP structure formation.

#### 2.3.1. Molecular Dynamics Simulations

The CG-MD simulations of HBcAg${}_{2}$ protein dimers were conducted using the Martini FF (version 2.2P) with polarizable water (PW) [22,71] in *Gromacs* [72,73] version 2020.1. The Particle mesh Ewald (PME) method [74] was used to account for electrostatic interactions. For all simulations, the ‘new’ parameter set for the Martini force-field was used [22]. All simulations were conducted at an isothermal-isobaric (NPT) ensemble at the temperature of 293 K and compressibility of $3\times 10^{-4}$ bar${}^{-1}$. All systems were charge neutralized and additional 150 mM sodium chloride added to fulfill capsid formation conditions [51]. The velocity-rescaling algorithm [75] was used as a thermostat for all simulations. Two macromolecules A and B, i.e., two HBcAg${}_{2}$ (see Figure 1), were placed at a specific starting configuration (relative position and orientation) centered in a triclinic box with at least the distance of 5.5 nm to the periodic boundary condition (PBC). A convergence study of PBC with at least 8 nm distance was performed and showed similar potential trends. A similar MD approach has been previously employed and validated by AA-MD for modeling of the pyruvate dehydrogenase complex (PDC) [76,77,78].

The simulations were conducted in four steps: two sets of energy minimization, equilibration, and production MD. Firstly, systems were solvated with normal Martini water and energy minimization was performed without PME for up to 100,000 steps. Secondly, Martini water was replaced by PW and another energy minimization with PME for up to 50,000 steps was performed. The steepest descend algorithm with a tolerance of 10,000 kJ/mol/nm was used for both minimizations. Thirdly, equilibration was performed using a reduced timestep of 5 fs for a total time of 50 ps. Position restraints were employed on carbon backbone atoms with a force constant of 1000 kJ/mol nm${}^{2}$. To avoid oscillations with the employed position restraints, the Berendsen barostat [79] with a coupling constant of 4 ps was used. Finally, production MD runs were performed for 0.6 ns with a timestep of 20 fs using PW, PME and a Parrinello-Rahman barostat [80,81] (coupling constant of 12 ps). Energies between all groups of components (A, B, PW, ions) were calculated every 400 fs and saved every 10 ps together with molecule trajectories.

MD simulations were performed for different initial positions and orientations relative between the two molecules. To avoid overlapping or entangled molecules, configurations with a minimum distance of $d_{\mathrm{coll}},\mathrm{full}=0.4$ nm between any two atoms of A and B were allowed. Molecular positions, orientations, and energies during 0.5–0.6 ns of MD runs were collected by fitting each molecule to its reference structure and averaged to construct intermolecular potentials. PBC box size dependent properties, e.g., the water potential, were compensated by calculation of the residual from a linear trend against the number of water molecules or the number of ions, depending on the type of a potential. All potentials were grouped, and Lennard-Jones and Coulomb contributions were added. This led overall to the following potential components: A-B, A-A + B-B, A-PW + B-PW, PW-PW, A-ions + B-ions, PW-ions, ions-ions, bonds, G96-angles, improper dihedral angles, Coulomb reciprocal. Note that ‘+’ indicates the addition, while ‘-’ indicates potential between two groups of components. Together with the interaction potentials between the molecules themselves (A-B), effects of the solvent, ions, bonded interaction, conformational changes, and long-range electrostatics were also captured. In order to account the symmetric configuration space, i.e., when molecules A and B are equal, the relative configurations were analyzed in both A-B and B-A fashion and, consequently, two data points were generated for each MD simulation.

#### 2.3.2. Spatial Descriptors

Several spatial descriptors, estimating intermolecular distances between molecules studied, were used for investigating spatial correlations, trend modeling, and interaction potential (see Section S2.1 in Supplementary Information (SI)). For the lower-dimensional A-B trend modeling, the minimum distance ($\delta_{m}$) between backbone atoms of molecules A and B was used, while the RMS deviation ($\delta_{r}$) between backbone atoms of B was used as a distance measure between two configurations (B-B). For the full configuration space between two molecules a six dimensional space (6D) of relative position x,y,z and orientation α,β,γ (under the assumption of a stable structure in the chosen process conditions) was used.

#### 2.3.3. Multivariant Estimation using Universal Kriging

To perform the multivariant estimation of the interaction potential from the set of MD simulations in a 6D configuration space, a Universal Kriging (UK) approach was implemented. It addresses the need to determine optimal (regarding minimum estimation variance) weights for the inference of a spatially distributed and correlated random variable in an arbitrarily dimensional space from a set of data points by linear combination. The estimation of the interaction potential, $U_{K}$, as the superposition of potential components *P* (see Section 2.3.1) consequently becomes

(1) $$U_{K}\left(\vec{x},\vec{\theta }\right)=\sum_{p=1}^{P}U_{K,p}\left(\vec{x},\vec{\theta }\right)=\sum_{p=1}^{P}\sum_{i=1}^{N}w_{p,i}U_{p,i}\left({\vec{x}}_{i},{\vec{\theta }}_{i}\right)\;,$$

where $\vec{x}$ and $\vec{\theta }$ are the position and orientation in interaction space, *N* is the number of data points used for estimation, and $w_{p,i}$ are the desired weights for linear combination of data points for potential component *p*. Each potential component is treated separately and, thus, in the following the index *p* is dropped. Note that typically only a subset of data points is used for the estimation at a given location called the local neighborhood. This is motivated by computational feasibility and additionally leads to an improved estimate by local estimation of the mean.

In contrast to, e.g., Simple Kriging [61,64], UK describes the underlying random variable as superpositioned by a systematic trend $\mu (\vec{x},\vec{\theta })$, which it can be decomposed of as

(2) $$U\left(\vec{x},\vec{\theta }\right)=\mu \left(\vec{x},\vec{\theta }\right)+R\left(\vec{x},\vec{\theta }\right)\;,$$

where *R* is the residual. Such behavior is present in the case of macromolecular interaction with a specific interaction potential at short distances between molecules and an asymptotically to zero going potential for large distances. The systematic trend is then modeled as a linear combination of *M* deterministic basic functions $f_{m}$ as

(3) $$\mu \left(\vec{x},\vec{\theta }\right)=\sum_{m=0}^{M}b_{m}f_{m}\left(\vec{x},\vec{\theta }\right)=\sum_{m=0}^{M}b_{m}f_{m}\left(\delta_{m}\;\right)\;,$$

which is simplified in the lower-dimensional space of the minimum distance $\delta_{m}$ (Section 2.3.2) due to the complexity of macromolecular interaction in 6D space and a physically reasonable description of decaying interaction. A set of basic functions, documented in SI Section S2.2, was used for UK here. The fitting was performed using weighted least-squares, as implemented in *Matplotlib* version 3.3.4 in *Python*. Weights were derived by inverse Gaussian weighting with a kernel width of $\delta_{r}$ = 2 nm to avoid bias due to sampling heterogeneity. The best resulting fit concerning $R^{2}$ was then chosen in combination with the constant function (local mean estimation) to describe the trend.

Following, the remaining residual *R* can be determined for each data point by subtraction of the modeled trend. Optimality of the UK estimate requires that the residual *R* of the underlying variable, i.e., statistic process, is intrinsically stationary with zero mean, as well as being Gaussian [61]. While the requirement of a zero mean is fulfilled in $\delta_{m}$ space and further improved in the full 6D space by local estimation of the mean through the local neighborhood of data points [64], intrinsic stationarity is, strictly speaking, not fulfilled: With increasing $\delta_{m}$ between molecules A and B, the distribution of *R* changes from a Gaussian distribution to a delta distribution of zero at large $\delta_{m}$, as it is expected for asymptotic interaction decay in molecular interaction. In the Kriging context, intrinsic stationarity is primarily important to model spatial continuity of the underlying statistical process, i.e., potential, through a (residual) variogram. Consequently, in order to rectify the issue, spatial continuity is modeled in sections for which intrinsic stationarity is reasonably fulfilled, including a Gaussian distribution at short (i.e., binding) distances. For this, the interaction space is split into five regions over the interaction range in $\delta_{m}$. In each region data points from the respective region, as well as adjacent regions, are used. Spatial continuity is then modeled in each section using a variogram defined as

(4) $$\gamma_{R}\left(\delta_{r}\;\right)=\frac{1}{2}Var\left(R\left(\left(\vec{x},\vec{\theta }\right)+\delta_{r}\;\right)-R\left(\vec{x},\vec{\theta }\right)\right)\approx \frac{1}{2|N(\delta_{r}\;)|}\sum_{N(\delta_{r}\;)}({\left(R\left({\vec{x}}_{i},{\vec{\theta }}_{i}\right)-R\left({\vec{x}}_{j},{\vec{\theta }}_{j}\right)\right)}^{2}\;,$$

employing the root-mean-square distance (RMSD), $\delta_{r}$, as a distance measure between two configurations (see Section 2.3.2). Due to the number of correlation samples, i.e., $>O(10^{10})$, direct fitting of the widely used variogram models (see Section S2.2 in SI) was not possible. Consequently, correlation samples were first binned over their $\delta_{r}$ distance up to a 4 nm cutoff within each region and the standard deviation in each bin was used as the uncertainty for weighted least-squares fitting of the variogram model.

Trend and variogram fitting was performed separately for all components of the potential in the molecular interaction, as both are different for each component. The optimal weights for unbiased and minimum estimation variance were calculated by solving the UK system at location ι [61,62,63,64]:

$$\left[\begin{array}{ccccccc} \gamma_{R}\left(\delta_{r,1-1}\right) & \cdots & \gamma_{R}\left(\delta_{r,1-N}\right) & 1 & f_{1}\left(\delta_{m,1}\right) & \cdots & f_{M}\left(\delta_{m,1}\right)\\ \vdots & \cdots & \vdots & \vdots & \vdots & \cdots & \vdots \\ \gamma_{R}\left(\delta_{r,N-1}\right) & \cdots & \gamma_{R}\left(\delta_{r,N-N}\right) & 1 & f_{1}\left(\delta_{m,N}\right) & \cdots & f_{M}\left(\delta_{m,N}\right)\\ 1 & \cdots & 1 & 0 & 0 & \cdots & 0\\ f_{1}\left(\delta_{m,1}\right) & \cdots & f_{1}\left(\delta_{m,N}\right) & 0 & 0 & \cdots & 0\\ \vdots & \cdots & \vdots & \vdots & \vdots & \cdots & \vdots \\ f_{M}\left(\delta_{m,1}\right) & \cdots & f_{M}\left(\delta_{m,N}\right) & 0 & 0 & \cdots & 0 \end{array}\right]\left[\begin{array}{c} w_{1}\\ \vdots \\ w_{N}\\ \lambda_{0}\\ \lambda_{1}\\ \vdots \\ \lambda_{M} \end{array}\right]=\left[\begin{array}{c} \gamma_{R}\left(\delta_{r,\iota -1}\right)\\ \vdots \\ \gamma_{R}\left(\delta_{r,\iota -N}\right)\\ 1\\ f_{1}\left(\delta_{m,\iota }\right)\\ \vdots \\ f_{M}\left(\delta_{m,\iota }\right) \end{array}\right]$$

Trend and variogram functions were normalized to ensure the same order of all matrix components [82]. Bi-diagonal divide and conquer singular value decomposition (SVD, with maximum factor of $10^{6}$ between eigenvalues using double precision) were employed to resolve ill-conditioned matrices resulting from, e.g., Gaussian variogram functions due to the zero slope at $\delta_{r}\;=0$ nm. The closest *N* data points from the estimation location with respect to $\delta_{r}$ out of the full data set were found using an incremental search algorithm. A convergence study on the number of required data points, *N*, between 100 and 1000 using the random HBcAg${}_{2}$ data set (see Section 3.1.1) showed that N=100 is sufficient to estimate the interaction field variance for iterative refinement and N=500 is required to estimate the interaction potential (see in Section S2.3 in SI, Table S1).

After solving the linear system of equations, the determined weights were used to calculate the potential estimate for each component using Equation (1), which were then superpositioned for all components to determine the overall interaction potential. The corresponding estimation variance for each potential component *p* was evaluated as

(5) $$\sigma_{K}^{2}\left({\vec{x}}_{\iota },{\vec{\theta }}_{\iota }\right)=\sum_{i=1}^{N}w_{i}\gamma_{R}\left(\delta_{r,\iota -i}\right)+\sum_{j=0}^{M}\lambda_{j}f_{j}\left({\vec{x}}_{\iota },{\vec{\theta }}_{\iota }\right)\;.$$

The presented UK approach provides a powerful method to determine the best linear unbiased estimate of the interaction potential for one relative position and orientation based on the sample data set. However, it is computationally too expensive to be performed during an MDEM simulation (see Figure 3). Therefore, the interaction potential fields in our work were saved in homogeneous grids and multi-grids (see details in Section S2.4). The advantage of using such homogeneous grids is the fast determination of forces and torques using a numerical gradient operation (see Section 2.5).

To generate the data set for potential field estimation, a two-step sampling process using MD simulations was derived. Initially, sampling was performed based on a systematic random strategy to generate a data set sufficient for statistical analysis. Later, iterative refinement was performed using a supervised learning strategy exploiting knowledge of variance, potential minima and maxima, as well as gradient maxima (for details see Section S2.5.1 in SI). Overall, 29 iterations were performed, leading to a total of 375,000 MD simulations.

#### 2.3.4. Molecular Collisions

Due to the fact that the MD model cannot reasonably capture colliding configurations, they are not sampled and Universal Kriging cannot provide an accurate estimate of the interaction potential. As determining an estimation through lower-scale models is not straightforward, an effective model was developed. It is based on the correlation between an increase in the interaction potential and number of colliding atoms caused by overlapping molecules. Additionally, molecular flexibility to avoid collisions is accounted by the distance of a configuration to the next MD data. Details of the molecular collisions model are provided in SI (Section S2.5), including an objective function for quantifying structural stability of a capsid.

#### 2.3.5. 2D Example of Kriging and Sampling Algorithm

**Figure 4:**
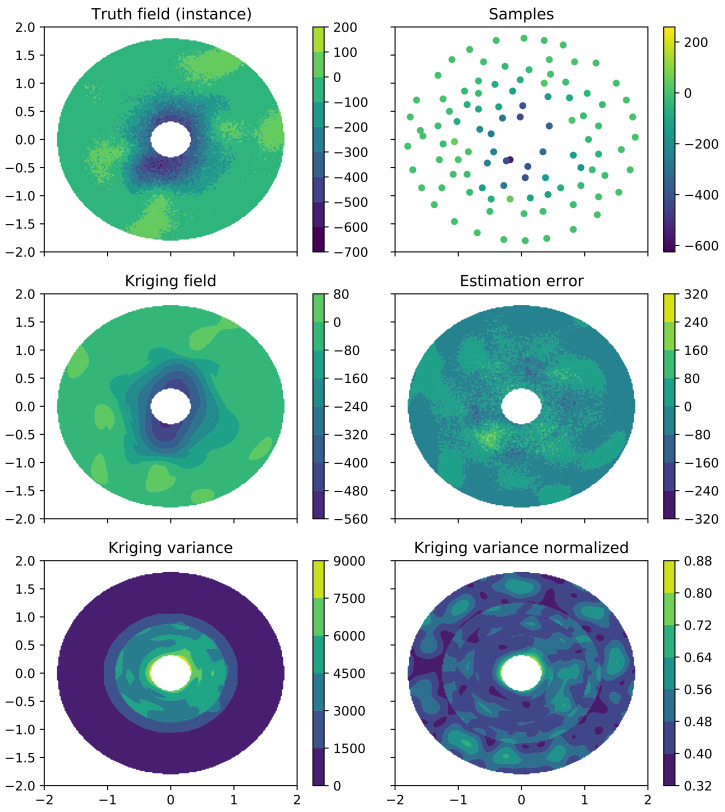
2D Universal Kriging example after 8 iterations with 10 samples per iteration and 20 initial samples (100 samples in total). For variogram determination the entire truth field was provided to ensure sufficient statistics. Test field possesses no units.

**Figure 5:**
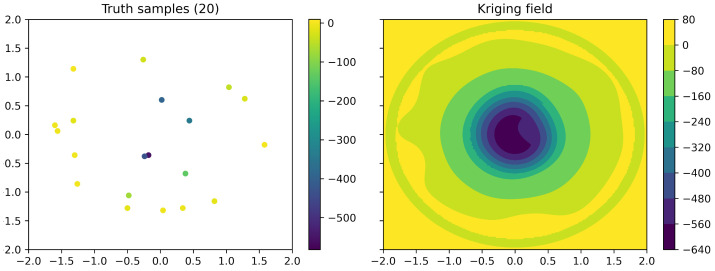

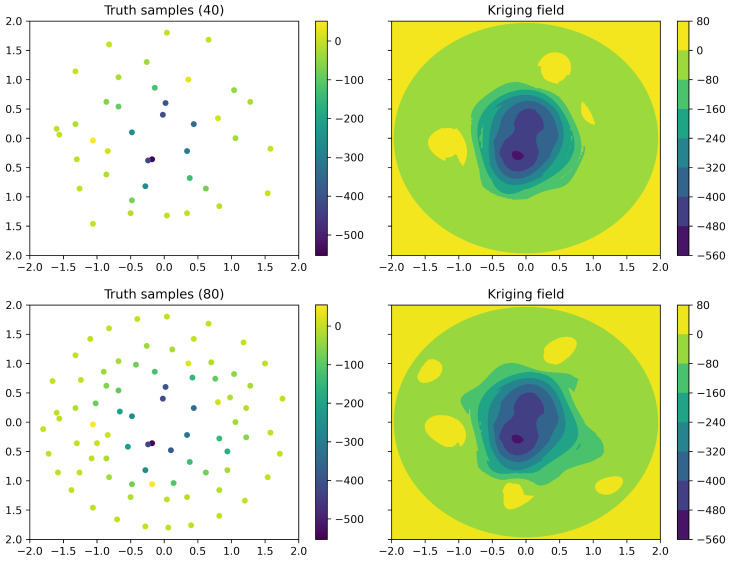
2D Universal Kriging example after initial random sampling (20 samples), two resampling iterations of 10 samples each (40 samples total), and six resampling iterations of 10 samples each (80 samples total). Test field possesses no units.

The algorithm explained was validated and visualized in a 2D simplified test case, representing the interaction between two single-atom molecules (see Figure 4 and Figure 5). The truth field of the 2D example was created as following: Firstly, a random field was created using sequential Gaussian simulation [83] with the spatial correlation described by a Gaussian variogram model with range r=0.7, nugget n=3000 and sill *s* = 10,000. Secondly, the random field was scaled to zero between a minimum distance of 0.4 and 1.2. Later, a trend over the minimum distance of Gaussian shape with −400 at minimum distance zero and range 1.0 was superpositioned. Such a test example possesses similar statistical properties as the molecular interaction data.

Based on this truth field (Figure 4), 20 initial samples are given to the Universal Kriging algorithm that has to ’learn’ the overall field through eight iterations of ten samples each. In this simplified case, only re-sampling through normalized variance minimization was performed. (Note that due to the small number of samples, the algorithm was given the entire field for variogram determination.) As it can be seen in Figure 5, the algorithm strategically places re-sampling points to reduce the overall variance and ‘learn’ the field. With each iteration, the field estimate improves and the remaining estimation error consists largely of small-scale discontinuities due to the inherent noise. In addition to this re-sampling based on the normalized variance, the main algorithm also performs more elaborate re-sampling based on identification of potential minima, maxima, gradient maxima, and absolute variance, to localize and quantify, e.g., binding locations. Details are provided in SI (Section S2.5.1). Furthermore, note the circular sections in the variance of Figure 4, indicating the separate variogram regions over the minimum distance that ensure intrinsic stationarity (see Section 2.3.3).

#### 2.3.6. Biased MD Sampling and Insertion of Empirical Data

Effective surrogate models relying on bottom-up parameterization, such as the one reported here, are often limited by the underlying lower-scale model. Due to finite sampling density, finite MD simulation time, as well as MD force-field accuracy and applicability, several limitations apply. These limitations are especially significant in the context of capturing binding events, which might be rare at the time scales addressable by MD, especially if they are connected with the conformational changes of molecules. To address these limitations in our model and improve the overall interaction potential, two effective modifications were added, preceded by the identification of the underlying reasons.

In the first approach, biased MD sampling was performed. It is known that during capsid assembly four binding locations between dimers are present. We extracted them from the reference capsid and placed the reference dimers at these relative configurations (see Table 1). The overlap of atoms was identified, which results from the conformational flexibility of capsid proteins and structural changes required for binding. Such intermolecular binding cannot be precisely captured by the underlying MD model, indicating the challenges of binding representation via MD. In order to perform biased simulations at these configurations, firstly the overlap was corrected: A search algorithm on the relative position and orientation (five steps of 0.2 nm or 10° in each direction) identified the closest overlap-free configuration measured by the RMSD of backbone atoms. A set of 1016 MD repetitions with 10 ns simulation time at each binding location was performed and its impact on the interaction potential was investigated.

In the second approach, the insertion of empirical data points was explored. Such an approach is especially useful if previous information on interaction (e.g., binding or repulsion) is present and meant to be incorporated into the interaction potential. It was implemented in our model by inserting virtual data points at the binding location between macromolecules into the data set (see Table 1). Those virtual data points only influence the nearby interaction potential and have no influence on overall potential trends and correlations. A variety of approaches were explored and the solution, leading to the most stable capsids (see Equation (S14) in SI Section S2.5 for stability measure), was applied. It is based on the insertion of two sets of data points as following: The first set with a constant potential $U_{\mathrm{bind}},\mathrm{center}$ is centered at the binding locations and replicated at 0.1 nm steps in each direction (rotational equivalent). The second set, representing a potential well (i.e., its shape), was located at increasing distance and potential from the binding location as a function of $\delta_{r}$ in all directions from $U_{\mathrm{bind}},\mathrm{center}$ to $U_{\mathrm{bind}},\mathrm{outer}$. The best solution found consists of a Gaussian potential of range $r_{\mathrm{bind}}$ (see Equation (S7) in SI) on a grid of −0.4, −0.2, 0.0, 0.2, 0.4 nm in all directions (rotational equivalent).

Inclusion of any virtual data point was restricted by two conditions: (a) the point should be within the range $r_{\mathrm{bind}}$, (b) the point should not result in more than 10 additional backbone collisions. Results of the approaches mentioned are presented in Section 3.

#### 2.3.7. Summary and Implementation

In summary, a data-driven methodology for deriving intermolecular interaction potentials from MD using Universal Kriging has been presented. The UK approach enables the best linear unbiased estimation of the interaction potential based on a set of data points and the presented iterative refinement enables supervised learning and improvement of the interaction potential in a near-optimal fashion. Overall, the proposed methodology consists of the following steps (as illustrated in Figure 3):

1. **Molecular reference structure** of all involved molecules from, e.g., a protein data bank. This reference structure has to be the same as used for the parameterization of the diffusion model [65].
2. **Initial interaction potential sampling** using MD and the outlined sampling methodology. (For large interaction spaces proximity sampling might be required for sufficient correlation data.)
3. **Trend fitting** in a lower dimensional interaction space of $\delta_{m}$ for all potential components.
4. **Correlation analysis and sectional variogram fitting** of trend-compensated residual *R* for all potential components. Identification of potential components with reasonable spatial continuity besides trend (only fulfilled by A-B potential).
5. **Grid design** based on interaction distance and memory size constraints.
6. **Universal Kriging** for multivariant estimation of interaction potential component residual *R*.
7. **Molecular collision** accounting as a function of molecular overlap and flexibility with increasing interaction potential.
8. **Iterative refinement** of field estimate based on estimation variance and extrema (potential minima/maxima, gradient maxima) localization and specification.

The framework has been implemented in custom C++/Python/Bash code with hybrid MPI+OpenMP parallelization. The various components were implemented in a semi-automatic fashion, including error checking and allowing for user supervision. The library *Eigen* (revision 14db78c53) was used for solving the linear system of equations of Universal Kriging and function fittings were performed using *Matplotlib* version 3.3.4 in *Python*. Potential fields were saved in floating precision within a custom binary format including the grid specifications.

Once the intermolecular interaction potential *U* is derived for all type permutations of molecules, a numerical gradient operation has to be performed to derive forces and torques on each molecule A resulting from an interacting molecule B at the relative position ${\vec{x}}_{body,A\to B}$ and orientation ${\vec{\theta }}_{body,A\to B}$ in the body frame of reference of A as

(6) $${\vec{F}}_{body,A\leftarrow B},{\vec{M}}_{body,A\leftarrow B}=-\nabla U\left({\vec{x}}_{body,A\to B},{\vec{\theta }}_{body,A\to B}\right).$$

Here, this gradient operation was performed using central differences (see details in Section S2.5.2 in SI). Due to the high dimensionality of the problem, gradient pre-calculation and saving was not feasible. While alternative representations, such as neural networks or functional fits, are possible, the general representation of the potential field and online gradient operation during the simulation was chosen for this work, as it provides the greatest flexibility and constant run-time.

### 2.4. Diffusion Model

Anisotropic diffusion of the abstracted macromolecules including the desired canonical ensemble was modeled using the previously reported diffusion model [65]. The determined diffusion coefficients for HBcAg${}_{2}$ are listed in Table 2. The parameterization was performed at 293 K and 150 mM of NaCl.

Due to the significant complexity in defining and solving the relative friction tensor for anisotropic molecules, such as the HBcAg${}_{2}$ dimer, hydrodynamic interaction was neglected. This is in agreement with the literature [67]; however, strictly speaking, this approximation is only fulfilled for dilute systems. During molecular self-assembly, as the solvent (water) around each macromolecule (in our case the HBcAg${}_{2}$ dimer) is replaced by other macromolecules, the friction and random forces in LD, resulting from the solvent, are reduced and the DOF between macromolecules become increasingly correlated. For example, a sphere surrounded by four equivalent spheres, positioned at 2.2 times the radius along the axes of a plus ’+’ (similar to the positioning on the VLP capsid), experiences only 5.8% of its normal drag force in direction of the surrounding spheres and 12.9% perpendicular to the plane formed by the spheres (approximated using Rotne-Prager-Yamakawa tensor [84,85]). Thus, the hydrodynamic interaction in our method was included in a simplified fashion, i.e., via a reduced effective viscosity of 10%. Similar approaches are well established in the literature: They often reduce the effective viscosity more significantly, i.e., by a factor between 10 and 1000 [32,86,87]. Such a decrease in effective viscosity additionally accelerates the dynamics of the system, while largely maintaining equilibrium [29]. Furthermore, note that the majority of intermolecular interaction during self-assembly, aimed to be captured here, is a result of the relative position and orientation between macromolecules, which is fully captured in the model developed.

### 2.5. Usage and Implementation within the Molecular Discrete Element Method

The discrete model for interaction of macromolecules was implemented in the open-source DEM code MUSEN [88]. The diffusion model and its implementation has been published in ref. [65]. The gradient operations for deriving forces and torques from the intermolecular interaction potential field are described in SI (Section S2.5.2). Unless otherwise specified, a temperature of 293 K was used and the corresponding dynamic viscosity of $1.0074\times 10^{-3}$ Pa s for water. The leap-frog algorithm was used for time integration and contact detection performed using a Verlet list implementation with an extended interaction radius derived from the intermolecular interaction distance for each molecule kind. Periodic boundary conditions were used throughout and unless otherwise indicated a time step of $10^{-13}$ s used. For details on the critical time step please refer to Section S2.5.3 in SI.

The models were implemented in C++ and CUDA (Toolkit v11.2 by NVIDIA [89]) for simulation on CPUs and GPUs, which are especially advantageous in the context of discrete simulations. Single precision was used throughout the code in contrast to the MUSEN default, which is sufficient in the context of the random component introduced by the diffusion model. Special emphasis was placed on kernel-level optimization of the numerical gradient operation, which is the most computationally intensive component. Additionally, helper fields indicating gradient-free locations within the grid were implemented to optionally speed-up computations. Code verification was performed using energy conservation analysis in artificial potential fields. Overall, a performance gain of approximately six orders of magnitude could be achieved between coarse-grained MD and the MDEM abstraction layer of entire macromolecules. This gain is primarily caused by the implicit solvent model, reduced number of degrees of freedom, and increased simulation time step. The proposed method thus enables investigation of entirely new phenomena and scales in comparison to traditional MD.

#### 2.5.1. Simulation Procedure

In order to assess the derived interaction potential in the context of the molecular system, three different areas were investigated. The respective simulation procedures are denoted as SPX.

##### VLP Binding Agreement and Stability (SP1)

**Figure 6:**
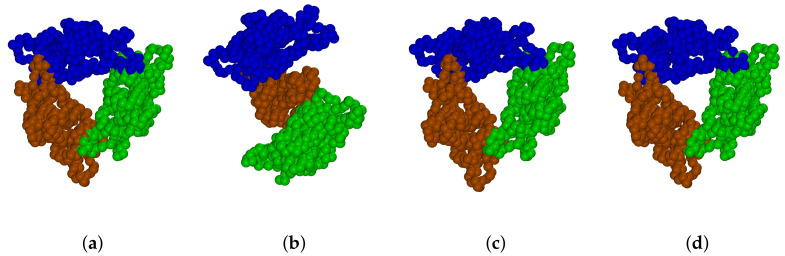
Visualization of trimer equilibrium conformations for various interaction potentials after equilibration (SP1): capsid reference conformation (**a**), pure MD-based potential (**b**) (Section 3.1.1), biased MD-based potential (**c**) (Section 3.1.2), with empirical data (**d**) (Section 3.1.3).

Firstly, the binding location agreement and stability was assessed based on a reference trimer of HBcAg${}_{2}$, extracted from the HBV capsid, which can be seen in Figure 6a. For this, a simulation at T = 0 K was performed for 25 ns enabling the system to equilibrate to the respective (local) potential minimum of the interaction potential field without interference of diffusion. Thus, binding location agreement and stability can be assessed and visualized on the smallest structural scale.

##### VLP Capsid Stability (SP2)

Secondly, capsid stability was assessed based on the reference capsid using the objective function in Equation (S14) (see Section S2.5 in SI) for quantitative evaluation of stability. For this, simulations at T = 293 K were performed for 250 ns and stability quantitatively evaluated at 1 ns intervals using Equation (S14).

##### VLP Self-Assembly (SP3)

Thirdly, VLP self-assembly was investigated based on a randomly initialized system of HBcAg${}_{2}$ dimers. For this, the dimers were placed at a random location and orientation in the simulation domain. Four different core protein concentrations of 5 μM, 10 μM, 50 μM, and 100 μM were investigated. In order to maintain comparable run times and statistics, the two lower concentrations were conducted in a 1 μm3 (1 μm edge) domain, while the higher concentrations were conducted in a 0.125 μm3 (0.5 μm edge) domain. After random placement, assembly simulations were performed for 5 ms (time step of $10^{-12}$ s and saving interval of 500 ns) at T = 293 K with a reduced viscosity of $1.0074\times 10^{-4}$ Pa s as discussed in Section 2.4.

#### 2.5.2. Postprocessing

The stability of pre-assembled capsids was quantified during SP2 (capsid stability analysis) using the objective function $O_{\mathrm{stab}}$ provided in Equation (S14) (see Section S2.5 in SI for definition). During self-assembly, structural formation was assessed using a network search algorithm differentiating between structured contacts (within a $\delta_{r}$ of 1 nm from a known binding location) and unstructured contacts (within a minimum distance $\delta_{m}$ of 0.3 nm and more than 1 nm from a known binding location). The size of each self-assembled structure (SAS) comprising of both contact types is quantified by the number of dimers/particles $N_{\mathrm{SAS}}$ and its diameter of gyration $d_{\mathrm{SAS}},\mathrm{gyr}$. Assembly kinetics are quantified by exponential fitting of the average of $N_{\mathrm{SAS}}$ over time (see Equation (S5) in SI with asymptotic value *N*_SAS,asymp_ (*s* in Equation (S5)) and time constant $\tau_{\mathrm{SAS}}$ (*r* in Equation (S5))). Additionally, the differentiation between structured and unstructured contacts is used to quantify the assembly quality by the average number of structured and unstructured connections per dimer $\xi_{\mathrm{struc}}$ and $\xi_{\mathrm{unstruc}}$, respectively. A perfect 120 mer capsid of HBcAg${}_{2}$ is characterized by $\xi_{\mathrm{struc}}=4$ and $\xi_{\mathrm{unstruc}}$ near zero. The fraction of structured contacts out of all contacts is termed $\Phi_{\mathrm{struc}}=\xi_{\mathrm{struc}}/\left(\xi_{\mathrm{struc}}+\xi_{\mathrm{unstruc}}\right)$. In addition to a global application, these measures can also be used on a per SAS or per dimer basis.

Moreover, the transition between size classes of $N_{\mathrm{SAS}}$ was tracked for each dimer at the discrete saving intervals of 500 ns to investigate the assembly mechanisms. The total number of transitions between all classes was normalized by the number of dimers in the system and visualized using chord diagrams employing the *circlize* library version 0.4.13 [90] in *R*. In addition to the total transitions between classes also the net transitions are provided in the SI, i.e., sum of both directions between classes. Based on this transition analysis, the lifetimes of structures $t_{\mathrm{life}}$ were additionally analyzed as defined by their duration of existence (up to at most the end of the simulation).

## 3. Results and Discussion

### 3.1. HBcAg${}_{2}$ Interaction Potential and VLP Stability

In the following section, the interaction potential between HBcAg${}_{2}$ dimers will be presented and its impact on VLP stability discussed in the context of trimer units as the smallest structural assembly of VLP (SP1). Three interaction potentials based on pure MD-based sampling, biased sampling at binding locations, and MD-based with inserted empirical data will be presented. The equilibrated trimers for each interaction potential can be found in Figure 6 and will be discussed in detail subsequently.

#### 3.1.1. Pure MD-Based Interaction Potential

The interaction potential between HBcAg${}_{2}$ dimer units was derived purely on the MD-based sampling and multivariant estimation using Kriging outlined in Section 2.3. Based on a set of pairwise MD simulations, randomly located at relative positions and orientations (see Table S2), the initial interaction potential was estimated and then iteratively refined (see Section S2.5.1). During resampling, the trend and variogram analysis are left flexibly to the algorithm and consequently change with each iteration.

##### MD Data

Statistical analysis of the (final) interaction data (see Section S3.3 in SI for full overview) shows an attractive trend for potential A-B, repulsive for potential A-PW + B-PW, attractive for potential PW-PW, repulsive for potential A-ion + B-ion, attractive for potential PW-ion (<5 kJ/mol), and repulsive for bond potential (<5 kJ/mol), while the potentials A-A + BB, ion-ion, G96-angles, improper dihedral angles, and Coulomb reciprocal contain no significant/valid trend. Consequently, conformational changes account only for a slight repulsive potential based on bonded interaction, but remain largely stable during inter-dimer interaction. Furthermore, long range electrostatic effects (Coulomb reciprocal) and interaction between ions appear negligible. Dominating factors of the interaction are found to be direct molecular interaction, solvent effects, and ion mediation. The sum of all minimum distance trends μ, i.e., without detailed residual potential *R*, has a local minimum of -32 kJ/mol at $\delta_{m}\;\approx 0.45$ nm and increases at $\delta_{m}\;=0$ nm back to approximately −9 kJ/mol (see Figure S4 in SI). This is slightly higher than experimentally reported association energies of HBcAg${}_{2}$ for HBV capsid assembly [54,91], where allostery effects modulating self-assembly are explicitly accounted, however, in accordance with other theoretical models [92,93]. After subtraction of the trend, out of all potential components only potential A-B contained a reasonable correlation between data points to employ Kriging. Variogram values varied between approx. 1000–10,000 kJ${}^{2}$/mol${}^{2}$ and correlation ranges between 2 and 4 nm depending on $\delta_{m}$ section. Other potential components contained significantly larger noise and/or very short correlation ranges, as it can be seen for all potential components in SI (Section S3.3 Figures S6–S16).

##### Convergence

**Figure 7:**
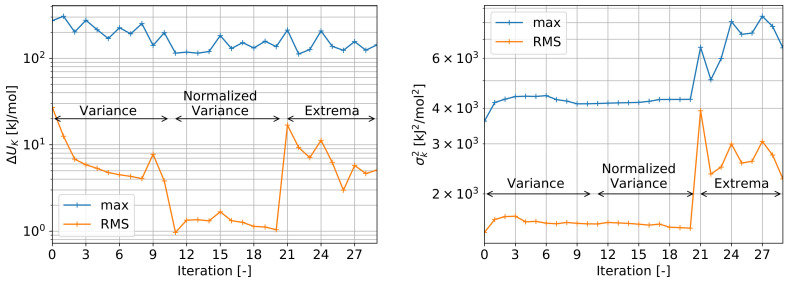
Convergence of the iterative resampling procedure for potential changes (**left**, $U_{i}-U_{i-1}$) and variance development (**right**). Figure adapted with permission from Ref. [66]. Copyright 2022, Springer.

Resulting from the supervised learning iterative resampling strategy (see Section S2.5.1 and Table S2), the interaction potential changed with each iteration and the resulting convergence plots can be found in Figure 7. ΔU indicates the potential change between consecutive iterations and $\sigma^{2}$ the field variance (both within cutoff and outside repulsion). The three main criteria for resampling (estimation variance, normalized estimation variance, extrema) can be clearly distinguished in the convergence plots and show a decreasing change in root-mean-square (RMS) potential changes over each resampling criteria. It can be noted that the resampling of extrema leads to higher changes in the RMS potential, which is attributed to the larger deviations from the trend of potential minima/maxima and gradient maxima being sampled. At the same time, the maximum change of the interaction potential decreases only slightly from approximately 300 kJ/mol to the range of 100–200 kJ/mol indicating large changes remain to occur locally with each iteration. Concerning the estimation variance, only a slight decrease in RMS estimation variance over the variance resampling region (iteration 1–20) can be seen. During extrema resampling (iteration 21–29), the estimation variance increases drastically at first, followed by a decrease in its RMS and stabilization concerning its maximum. This drastic increase is attributed to the increased variance near extrema locations and consequent impact on the variogram model. Stability of the maximum change in potential for iteration 21–29 indicates that these extrema samples contain primarily a larger variance. Overall, while changes in interaction potential decrease, convergence remains challenging due to the dimensionality of the interaction space as well as inherent noise.

##### Resulting Field

**Figure 8:**
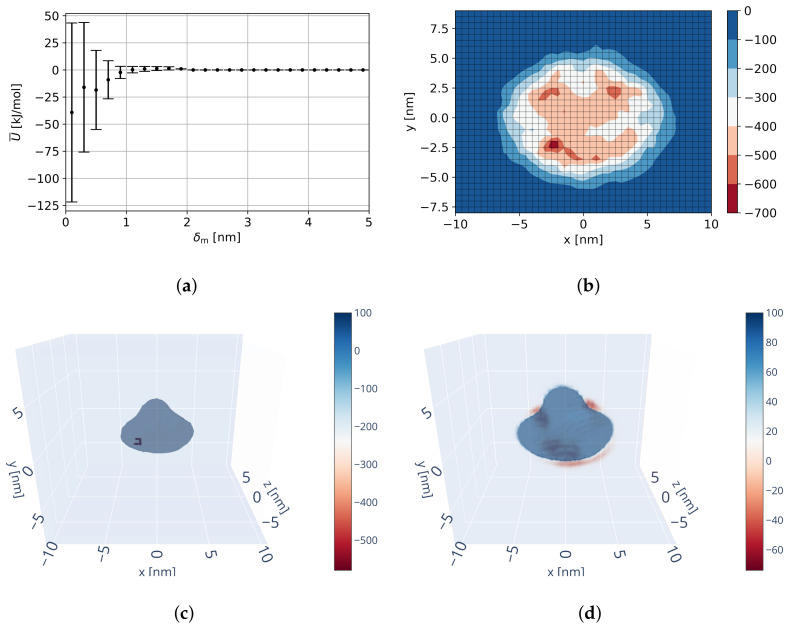
Visualizations of the potential field based on pure MD-based sampling strategy. (**a**) Grid average and standard deviation binned over minimum distance. (**b**) X-Y cross-section minimum over all remaining dimensions. (**c**) 3D minimum over orientations. (**d**) 3D mean over orientations. (**a**,**b**) adapted from with permission from Ref. [66]. Copyright 2022, Springer.

The resulting overall interaction potential is visualized in Figure 8. As Figure 8a shows, the interaction range is approximately 2 nm in $\delta_{m}$ and prior to an attractive behavior a potential barrier of approx. 0–5 kJ/mol at $\delta_{m}\;$ ≈ 1.5 nm has to be overcome. The potential minimum at $\delta_{m}\;$ ≈ 0.45 nm resulting from the trend is slightly increased when averaged over all grid locations and the binding potential at $\delta_{m}\;=0.45$ nm decreased. Furthermore, it can be seen that the field variance (not estimation variance) increases with decreasing distance between molecules indicating both binding and repulsion at short distances depending on relative configuration. These binding potentials are significantly lower in value than those of the trend itself, which is attributed to strong electrostatic and van der Waals interaction at short distances.

As it can be seen in the 2D minimum projection in Figure 8b and 3D minimum/mean in Figure 8c,d, the identified interaction potential contains three main binding locations: in negative and positive x-direction next to the dimer spike (positive y-direction), as well as underneath the dimer (negative y-direction). When performing equilibration of the reference trimer with this field (see Figure 6b), the dimers are pushed to be located with their underneath (negative y) next to the spike of its interaction partner. These identified binding locations are notably different from the expected binding locations (see Figure 6a and Table 1). As a result, the derived interaction potential does not produce stable capsids.

Upon investigation of the underlying data and details of binding, these differences and limitations (in capturing binding) are attributed to the conformation of the reference structure with respect to that of the binding conformation (self-assembly of viruses is a highly allostery-driven process), MD timescales, and possibly the employed Martini force-field. As it can be seen in the overlapping side-chains in Figure 6a, the reference structure, derived from the representative clustering with the force-field used, deviates from the conformation during binding, which results in overlapping molecules at the binding configurations. While structures possess no additional constrains during each MD run and binding at these relative configurations is allowed, allostery-induced conformational changes at the binding locations, occurring during real self-assembly, cannot be fully captured by the unbiased simulation. In order to improve the sampling and conformational challenges during binding, extended MD simulations near the binding locations are performed next. Note that testing alternative force-fields goes beyond the scope and computational capabilities of this work.

#### 3.1.2. Biased MD Interaction Potential

**Figure 9:**
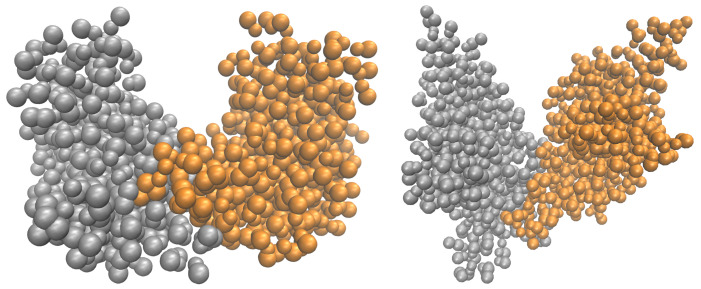
Visualization of two interacting HBcAg${}_{2}$ obtained from the biased MD simulation with lowest potential A-B (side view left, top view right).

Results of biased MD simulations indicate improved binding recognition, but remaining low probability of strong binding (i.e., low potentials) as well as remaining conformational differences at the flexible C-terminal region, although overall conformationally similar to literature [43,56,94]. The intermolecular potential A-B over all replicas and all four binding configurations has a minimum potential of −762 kJ/mol indicating stronger binding than the previous potential field (Figure 8), but the average potential remains at −285 kJ/mol, as well as the largest replica fraction around −350 kJ/mol. Consequently, probability of binding to occur remains low. The binding configuration of the lowest potential is visualized in Figure 9 and resembles expectations from the literature [43,56,94].

The resulting interaction potential after inclusion of data from biased MD simulations shows no notable visual differences and can be found in SI (Figure S5). Note that trend and variogram models were generated without biased data. In comparison to the purely MD-based field (over all grid locations in the interaction range), the average potential decreases slightly by −0.05 kJ/mol and is locally lowered by up to −299 kJ/mol as well as increased by up to 234 kJ/mol. These local changes appear to improve the binding location, nonetheless, as this potential was found to keep the reference trimer stable during field equilibration (see Figure 6c). This improvement is quite significant and underlines that binding recognition appears to be a major issue at this point. However, the biased interaction potential remains unable to keep the capsid stable as global binding locations remain unchanged to that of the pure MD potential. Consequently, binding probability remains too low and differences in capturing the conformational changes of residues in the C-terminal remain, which cause the interaction potential at the binding location to be not specific and strong enough.

Such limitations of lower-scale models are well known in force-field and effective surrogate model development [29]. In many cases additional (external) knowledge is necessary to improve the effective model (in this case interaction potential) at strategic locations or coarse-grained force-fields employ an entirely top-down parameterization approach (e.g., Martini [23]). In the following section, a hybrid approach is explored.

#### 3.1.3. MD-Based Interaction Potential with Empirical Data

After performing several tests on inserting empirical data of binding locations, we have found that to generate reasonably stable capsids the binding potential has to be lower than the potential minima of the pure MD-based potential (at least −800 to −1000 kJ/mol at binding location) and binding shape has to be approximately 1 nm in range with a Gaussian profile of increasing potential. With decreasing potentials to the range of −1400 kJ/mol, the capsids were found to improve in stability and self-assembly. Higher potentials were found to not be specific enough in contrast to the pure MD-based minima and wider potentials were found to be not spatially specific enough. While these potentials are very low, they are in agreement with binding occurring during biased MD simulations, especially in the context of remaining C-terminal binding conformational changes to occur [95].

**Figure 10:**
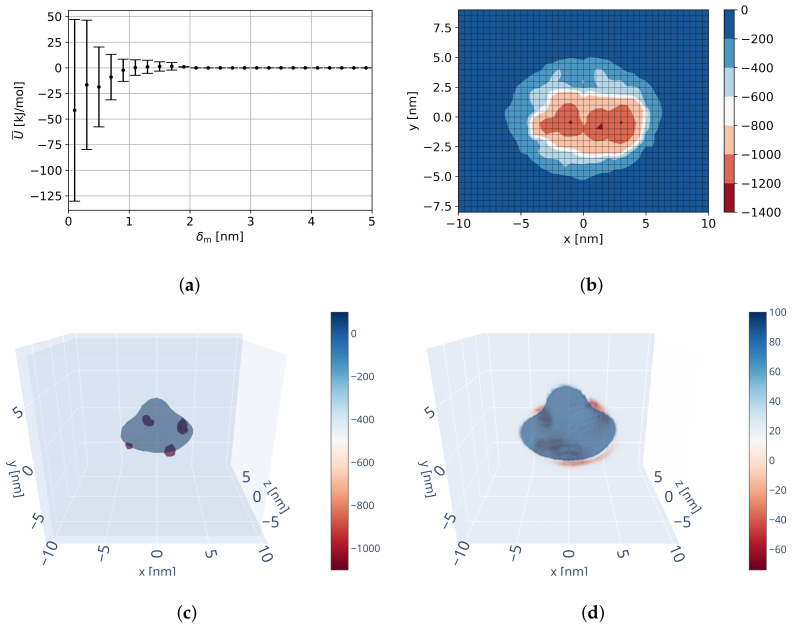
Visualizations of the potential field based on MD with inserted empirical data. (**a**) Average and standard deviation binned over minimum distance in grid. (**b**) X-Y cross-section minimum over all remaining dimensions. (**c**) 3D minimum over orientations. (**d**) 3D mean over orientations.

The best solution found concerning capsid stability and assembly (employing simulation procedure SP2, see Section 2.5.1) was able to keep the capsid stable with an objective function of $O_{\mathrm{stab}}=0.725$, which is a near perfect capsid. Virtual data points were inserted as specified in Section 2.3.6 with *U*_bind,center_ = −1400 kJ/mol, *U*_bind,outer_ = −1000 kJ/mol, and $r_{\mathrm{bind}}=1.0$ nm. As it can be seen in Figure 10, in comparison to the pure MD-based potential (Figure 8) the inserted virtual data points create new minima at the binding locations, but do not affect the remaining overall potential. This is important as remaining characteristics, such as the potential barrier at $\delta_{m}\;\approx 1.5$ nm, are kept and consequently knowledge from MD and empirical data are merged.

### 3.2. VLP Self-Assembly

**Figure 11:**
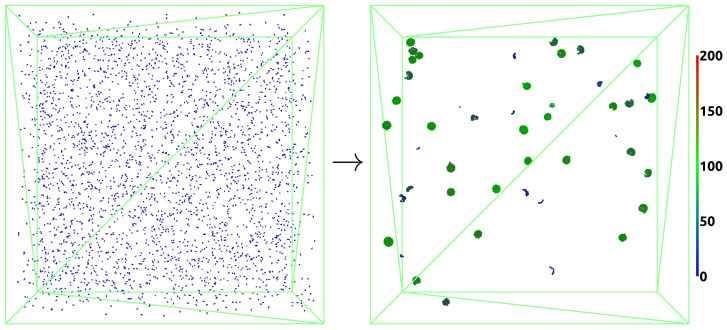
Self-assembly of VLPs during 5 ms simulated using MDEM with SP3 simulation protocol (box of 1 μm^3^, protein concentration of 5 μM). The size of assemblies formed ($N_{\mathrm{SAS}}$) is depicted using the designed color scheme and the backbone carbon atom representation.

We present the self-assembly process of virus-like particles from HBcAg${}_{2}$ dimer units based on the overall framework (depicted in Figure 3) with diffusion and the MD-based interaction potential that includes empirical data (Section 3.1.3). Four HBcAg${}_{2}$ concentrations of 5 μM, 10 μM, 50 μM, and 100 μM were studied at the ion concentration of 150 mM sodium chloride used for model parameterization, thus covering a wide range of conditions. Simulations of each system started from a random state (e.g., as shown in Figure 11), resulting in capsid formation, as well as other aggregates and intermediates, through self-assembly over the course of the simulation. In the following sections, we discuss properties of the capsids formed, including assembly kinetics and assembly pathways.

#### 3.2.1. Assembly Properties

**Figure 12:**
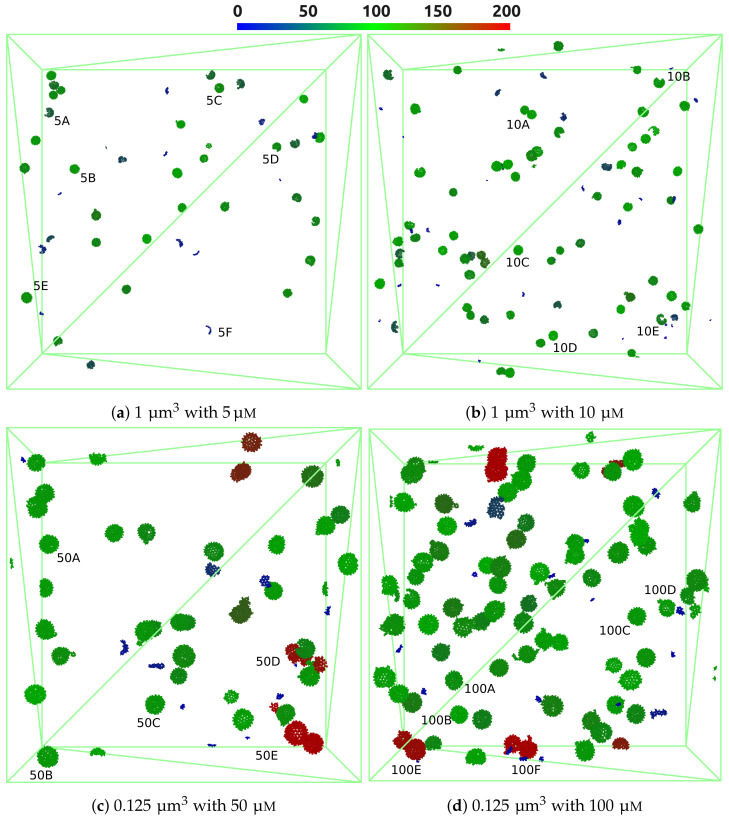
Visualizations of VLP self-assembly using simulation protocol SP3. Colors indicate structure size by number of dimers ($N_{\mathrm{SAS}}$) and backbone carbon atoms are visualized. Structure 50E exceeds scale with 221 and red structure at top left of (**d**) contains 233 dimers.

**Figure 13:**
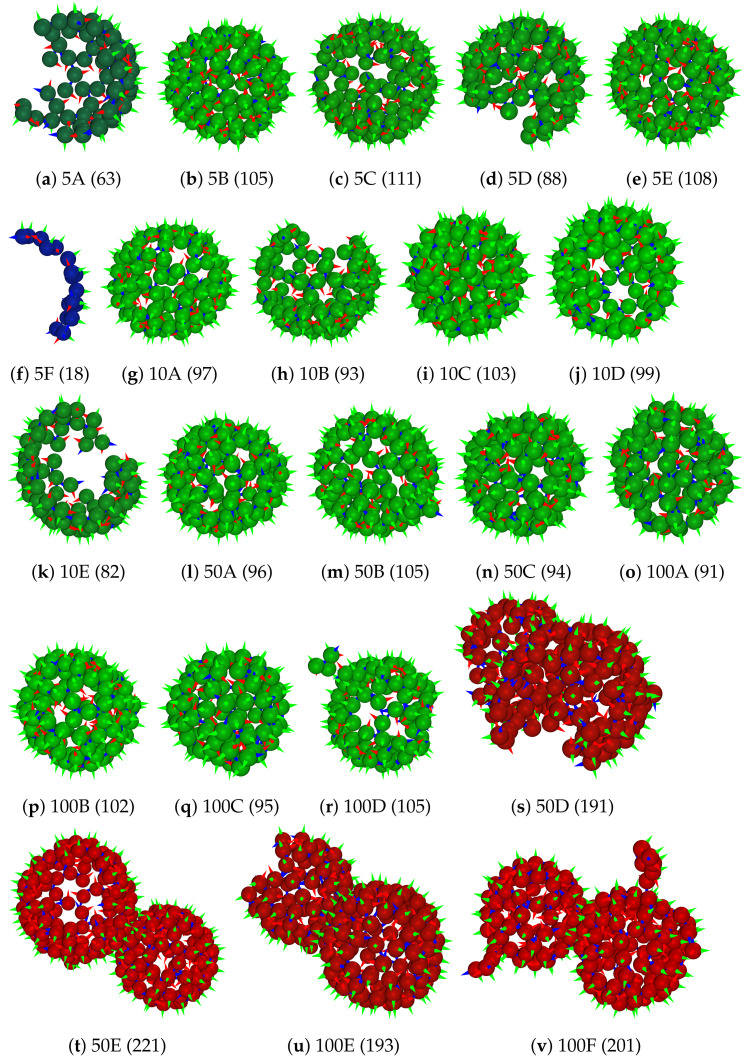
Magnified capsids marked in Figure 12 using visualization of dimers as spheres with orientation arrows (*x*-axis red, *y* green, *z* blue). Numbers behind identifier indicate $N_{\mathrm{SAS}}$ of structure. Colors match original coloring scheme according to $N_{\mathrm{SAS}}$ in Figure 12.

As can be seen in the visualization of all systems studied (Figure 12) and closeups of structures formed (Figure 13), the systems self-assembled from a random state primarily to spherical capsid structures around 100 dimers in size (green color). The capsid structures agree visually well with icosahedral expectations of structures for the majority of the population (see, e.g., Figure 13b,c,e). This is further supported by an average of $\xi_{\mathrm{struc}}=3.5$ structured connections per dimer for all concentrations (see Figure S1 in SI), which is close to that of the perfect T = 4 capsid (120 dimers) with $\xi_{\mathrm{struc}}=4.0$. However, equilibration appears to be incomplete and many capsids show defects with regard to missing dimers or dimer segments, as well as minor misalignments (see, e.g., Figure 13a,d,h,k). These defects are caused by the low availability of individual dimers (and small dimer assemblies) with advancing self-assembly. This is a well-known phenomenon also reported in experiments [54,96]. Significantly longer simulation times (beyond current computational capabilities) or addition of new individual dimers are likely required for the finalization to perfect T = 4 capsids with 120 dimers.

When visually comparing VLP formation in different concentrations, it can be seen that the primary population, comprising around 100 dimers, is similar for all concentrations (green structures in Figure 12). However, significant differences with regard to smaller and larger structures are detected and further discussed in Section 3.2.3. At lower HBcAg${}_{2}$ concentrations the number of smaller structures is higher, which can be considered as a pre-stage of capsids (see, e.g., Figure 13f) and highlight the diffusion limitation for the formation of larger assemblies. In contrast, at higher concentrations the number of overgrown (more than 120 dimers) capsids and colliding structures (i.e., temporarily touching otherwise intact capsids) is increased. While many still resemble correct icosahedral-like structures (see, e.g., Figure 13t–v), some also show more significant defects (see, e.g., Figure 13s). This is further highlighted by an increase in unstructured connections from $\xi_{\mathrm{unstruc}}=0.34$ for 5 μM to $\xi_{\mathrm{unstruc}}=0.42$ for 100 μM (see Figure S1 in SI). This modulation of capsid assembly by the initial concentration of core proteins known from experiments, i.e., higher tendency for kinetic traps and overgrown or aggregated capsids at higher concentrations [50,54,97], is therefore correctly represented by our multiscale model.

**Figure 14:**
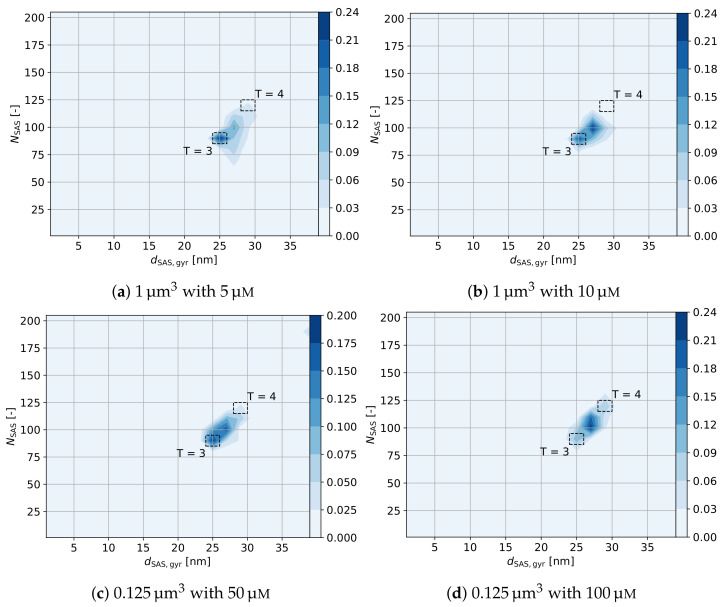
Final distribution of numbered size versus diameter of gyration (averaged over last ten saving steps).

The diameter of gyration relative to the number of HBcAg${}_{2}$ forming each capsid is depicted in Figure 14. It includes a marking of capsids with T = 3 and T = 4 symmetries, enabling further characterization of the self-assembled capsids. As it can be seen, all four concentrations show similar properties with regard to the primary populations. A smaller portion of the self-assembled capsids belongs to the T = 3 population made out of 90 dimers (i.e., 24.0%, 20.7%, 19.2%, and 11.5% with increasing concentration, respectively), while the majority of capsids can be considered as pre-stages of the T = 4 capsid with 120 dimers. These pre-stages miss approximately 10–20 dimers, while already closely resembling the final capsid as indicated by the diameter of gyration in addition to the structuredness of pairwise contacts ($\xi_{\mathrm{struc}}=3.5$). This excess of T = 4 capsids over T = 3 capsids is in agreement with literature [56,98,99,100] with more than 90% of T = 4 capsids expected. Overall, the self-assembled aggregates are highly structured and closely resemble the expected HBV VLPs.

#### 3.2.2. Assembly Kinetics

**Figure 15:**
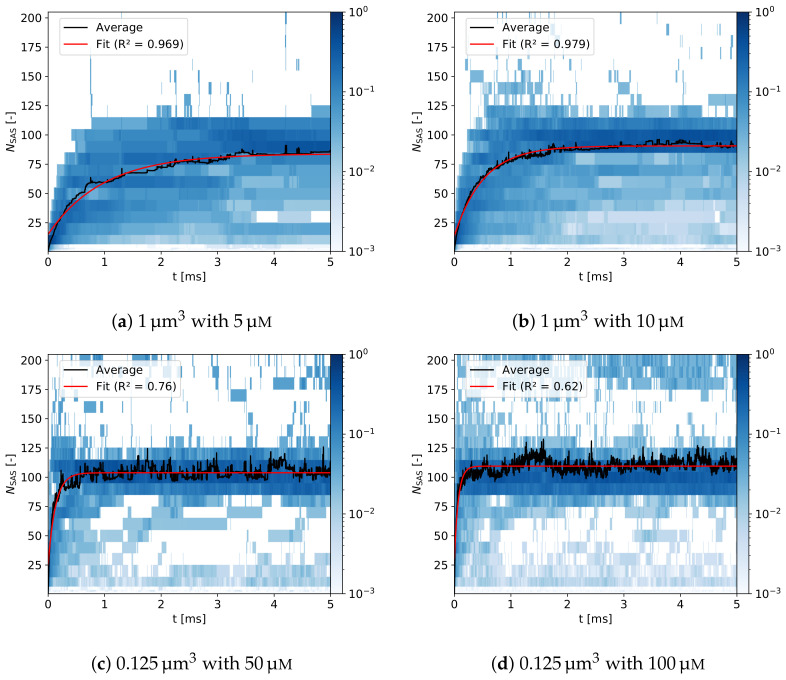
Histogram of self-assembled structures by number of constituting HBcAg_2_ (*N*_SAS_) over time. τ_SAS_ for concentrations in increasing order: 2.91, 1.55, 0.39, 0.21 ms. *N*_SAS,asymp_ for concentrations in increasing order: 83.9, 90.7, 103.8, 109.5.

For the first time, the multiscale model, developed in the present study, permits investigation of the VLP assembly in silico from the smallest building unit of the capsid, i.e., HBcAg${}_{2}$. The kinetics of capsid assembly for different protein concentrations are shown in Figure 15, where histograms of the size of self-assembled structures over the simulation time (5 ms) are demonstrated. As discussed above, all simulations result in the primary capsid population comprising around 100 dimers. This is more pronounced at the protein concentration of 10 μM. After the formation of this population, the equilibration significantly slows down leading to increasing time scales for finalization of perfect T = 4 capsids. This is not surprising since efficient self-assembly with the formation of correct capsids takes from several seconds to days in experiments [50,96,101], which is far beyond affordable simulation times. At the same time, capsids with the number of HBcAg${}_{2}$ in the range of 100–120 are more populated at slightly higher concentrations (see Figure 15c,d), which also supports experimental observations [52,97]. Similarly to the visual comparison of assembled systems in Figure 12, the growth of the capsids depicted in Figure 15 indicates an increased fraction of smaller structures (i.e., below 90 dimers) for low concentrations (i.e., 29.0% for 5 μM with a decrease to 7.5% for 100 μM) and an increased fraction of large structures above 120 dimers for high concentrations. This change in distribution additionally increases the asymptotic average structure size at the end of the simulation from 83.9 for 5 μM to 109.5 for 100 μM, respectively, (see description given in the caption of Figure 15).

From the observations described, we conclude that the final (average) size of the VLPs formed can be attributed to the diffusion limitation at lower concentrations [54] (resulting from increased mean distances of structures in the solution) and overgrowing or capsid collisions at higher concentrations [52]. Moreover, the large structures at high concentrations, especially for 100 μM, appear to undergo frequent transitions between population sizes and coincidentally contribute to $\xi_{\mathrm{unstruc}}$ increasing to 0.42 (from 0.34 for 5 μM, see Figure S1 in SI), thus causing an increase in unstructuredness of assemblies. Similar observations were summarized in the recent review by Bruinsma et al. [102].

The diffusion limitation at low concentrations is further highlighted by the longer equilibration times for such systems extending to $\tau_{\mathrm{SAS}}=2.9$ ms for a 5 μM concentration, see Figure 15. Please note that due to the accelerated dynamics of such coarse-grained simulations these time scales are only of comparative nature and not real-world time scales. In contrast, at the highest concentration of 100 μM the equilibration time is faster by more than an order of magnitude with only $\tau_{\mathrm{SAS}}=0.2$ ms. Similarly, the number of individual dimers in solution decreases to below 1% within 40 μs for 5 μM and only 2 μs for 100 μM, thus scaling inversely with the concentration. With regard to the functional relationship of assembly kinetics, the average structure size strongly follows an asymptotic exponential behavior throughout the concentration range (see fits in Figure 15 using Equation (S5) in SI). This is in accordance with current state-of-the-art [102,103,104,105], including calculations made using dodecahedral model with a trimeric nucleus of core proteins [106]. Alternatively, assembly kinetics can be analyzed by the average number of structured $\xi_{\mathrm{struc}}$ and unstructured $\xi_{\mathrm{unstruc}}$ contacts per dimer, which is provided in SI (Figure S1).

#### 3.2.3. Assembly Pathways

An important aspect, explored intensively during the last two decades, is understanding the pathways of virus self-assembly (and disassembly) that permits, on the on hand, to develop antivirals and therapies for treatments and, on the other hand, to design and predict new vaccines based on VLPs. Even if experimental investigations shined light on various phenomena, they have limited prediction power (also using rational design) that can be applied to estimate the self-assembly of previously not investigated capsid proteins, e.g., after mutations or other amino acid modifications used for chimeric VLP fabrication [101,107,108]. Here, computational approaches are highly demanded, but are also limited as we described in the introduction. With the multiscale method developed, molecular modifications in core proteins can be captured and explicitly accounted for in the changes of capsid self-assembly. In the following, we describe the pathways for wild-type HBV VLP self-assembly, obtained in the present study, and show its high potential to reproduce experimental observations.

**Figure 16:**
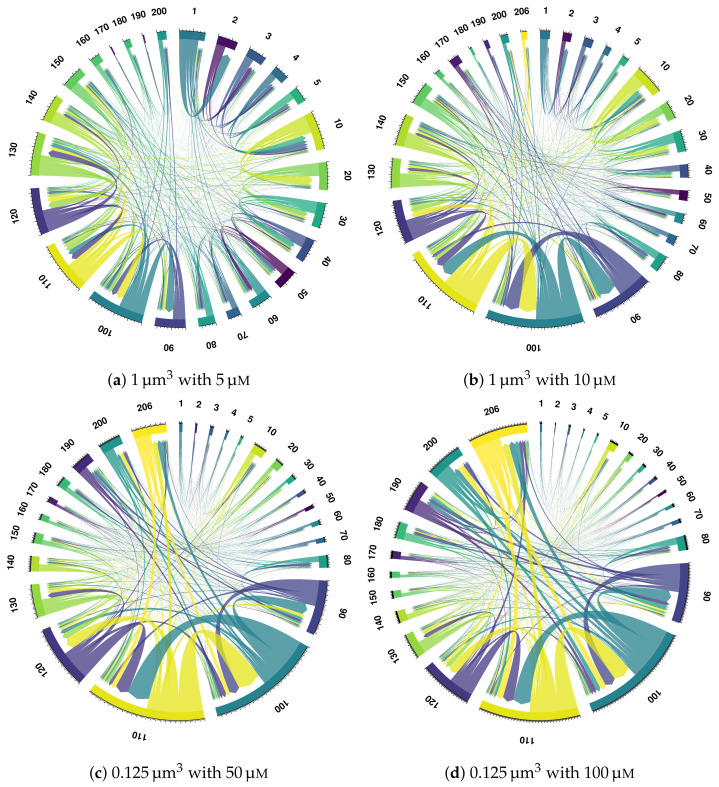
Self-assembly by bi-directional transitions between size classes normalized by total number of dimers (major ticks represent unit arrow thickness, i.e., every dimer makes this transition on average). Starting at class 10, the size denotes the class range between −4 to +5 relative to the noted value; 206 incorporates all sizes equal to or larger than 206. Colors provide contrast only. See Section 2.5.2 for further specifications.

Capsid assembly is known to be modulated by lots of weak interactions between its building units and to be characterize by multiple assembly pathways [50,91], which highly depend on experimental conditions, e.g., ionic strength or protein concentration. In order to visualize assembly pathways occurring during 5 ms MDEM simulation of HBV core proteins, chord diagrams were employed, which are shown in Figure 16. These chord diagrams incorporate all bi-directional transitions between different population classes, i.e., assembly sizes, normalized by the total number of HBcAg${}_{2}$. Net transitions, i.e., sums of both directions (assembly and disassembly), are additionally visualized in SI (Figure S2).

The complexity of self-assembly and existence of different pathways, including transition probabilities and types of pre-capsid structures, is clearly seen in Figure 16. Moreover, a hierarchical structural build-up in stages from smaller to larger capsid-like assemblies (especially using 5 μM solutions) is noticeable. The initially available HBcAg${}_{2}$ dimers (denoted as ‘1’, the unit structures used in the model) self-assemble into structures of two, three, four, five, and ten (6–15 range) with decreasing transition fraction in their first step. Larger assemblies (mostly up to 35-mers, see light green transitions from ‘10’-mer population to ‘20’-mer, i.e., 16–25 of dimers, and ‘30’-mer, i.e., 26–35 of dimers), are mostly built from these smaller ‘10’-mer structures (see additionally Figure S2 in SI). This is an interesting observation, which was recently reported experimentally [91].

With increasing concentration of HBcAg${}_{2}$ this assembly process accelerates (see Figure 15), further leading to a different transition distribution of dimers (‘1’) with an emphasis on a direct jump to ’10‘-mer structures (i.e., 6–15 of HBcAg${}_{2}$) within 500 ns at 100 μM concentration. Even if protein concentration increases with the visible rise in the number of intermediates and large assemblies, an important role of ’10’-mer population in the overall self-assembly is still visible. Moreover, its maximum lifetime (around 3.5 ms at 5 μM, see Figure S3b in SI) is higher than smaller assemblies and is in the range of more stable pre-capsid structures.

A stage-wise assembly through addition of smaller structures occurs until mostly the ‘80’-mer population, as indicated by the low direct transition rates to larger structures (visually arrows towards the center of the diagram). Larger capsid-like assemblies, e.g., ‘90’-mer population, are growing via partial disassembly from the overgrown structures (see green arrows from ‘100’-mer population, i.e., 96–105 dimers, in Figure 15). At the same time, the ‘120’-mer population shows two possible formation pathways, i.e., via overgrowth and a step-wise growth. This is especially visible at higher concentrations starting from 10 μM (see, e.g., high contribution of transitions from the ‘110’-mer to ‘120’-mer population (arrow in yellow) in Figure 15b–d and multiple transitions from higher assembly aggregates). The role of structural overgrowth during wild-type HBV capsid formation was shown by Lutomski et al. [50]. In addition, huge amounts of intermediates with 96–105 (‘100’-mers, e.g., structures 5B, 10A, 50A, and 100B in Figure 13) and 106–115 dimers (‘110’-mers, e.g., structures 5C and 5E in Figure 13) are visible in all chord diagrams. Similar intermediates (104/105-mer, e.g., 5B and 50B in Figure 13, and 110/111-mer, e.g., 5C in Figure 13) were proven also experimentally [53].

For all concentrations, the majority of transitions occurs around the class sizes between 90 and 120, thus being either T = 3 capsids or pre-stages of T = 4 capsids, as previously discussed. Consequently, this population region can be considered as semi-stable with transitions primarily motivated by a partial disassembly and re-organization with attempted addition of small, hopefully in proximity available, assemblies for the stable structure formation. However, there are also stable T = 3 capsids formed (see Figure 15 and Figure S3 in SI), suggesting both assembly and disassembly processes towards the formation of both types of VLPs, i.e., with 90 and 120 HBcAg${}_{2}$. Such behavior was reported to be extremely sensitive to experimental conditions [91] and is observed in the simulation here. As denoted earlier, the assembly to a perfect T = 4 capsid is mostly the question of probability and simulation time, especially in the context of how many small structures are available at a specific point of the self-assembly.

Above these size classes of 120 HBcAg${}_{2}$, structures undergo an increasing number of transitions on their pathway to equilibrium, which increases drastically in frequency and number with increasing concentration. While only few dimers form structures above 120–140 for low concentrations of 5 μM and 10 μM, the majority of dimers undergo such transitions for the larger concentrations of 50 μM and 100 μM indicating a pathway through overgrowth. However, it should be noted that these transitions also incorporate mere contacts of otherwise proper capsids, as shown in the visual inspection prior. Furthermore, structures above 120 dimers cannot be considered stable as they break apart on very short time scales as can be seen in Figure 15.

The assembly pathway and kinetics can additionally be recognized in the development of structure lifetimes $t_{\mathrm{life}}$, which is provided in SI (Figure S3). With increasing size, the average lifetime increases from the microsecond scale to the order of tenth of milliseconds for ’70’–’100’-mers before dropping back to microseconds above ‘120’-mers. This indicates instability of overgrown structures and stability of the region between T = 3 and T = 4 capsids. Similarly, maximum lifetimes increase to multiple milliseconds (up to the simulation time of 5 ms) for the range of ‘10’–‘120’-mers. Additionally, in the range of ‘90’–‘120’-mers the average lifetime decreases with increasing size supporting the previously attributed re-organization and finalization of T = 4 capsid structures. Lastly, with increasing concentration from 5 μM to 100 μM the average lifetime in the range ‘90’–‘110’-mers decreases drastically by one order of magnitude highlighting the increased number of contacts and unstructuredness of capsids at high concentrations. Moreover, the increase in kinetic traps and aggregated capsids is clearly captured in the simulation (see Figure 16), which agrees well with VLP yields obtained experimentally [50,54,97].

## 4. Conclusions

We have developed a generally applicable modeling framework based on a hierarchical coarse-grained strategy for capturing macromolecular self-assembly on scales beyond traditional MD. For this, each macromolecule is abstracted as an anisotropic object and high-dimensional data-driven models are generically formulated to describe interaction between molecules and with the solvent environment. As a result, the self-assembly process is described as a combination of diffusive effects and pairwise interaction of molecules, including effects of, e.g., dissolved ions. A Kriging-based strategy building upon high-throughput MD simulations with the Martini force-field is employed including semi-automated supervised learning to derive data-driven protein–protein interaction potentials. Through this approach, the multiscale method enables the significant speedup to the micrometer and millisecond scale, while maintaining the necessary high detail of intermolecular interaction in their 6D structure.

The framework was applied to study the self-assembly of hepatitis B virus-like particles starting from their minimal building unit, i.e., dimer of HBcAg. MDEM simulations of VLP formation were performed using four different protein concentrations (5 μM, 10 μM, 50 μM, 100 μM) at 150 mM NaCl. Differences in the formation of pre-capsids structures and their intermediates were analyzed. VLP formation as a hierarchial build-up and an overgrowing was captured. The key role of assemblies made out of 10 HBcAg dimers and up to 35 HBcAg dimers has been demonstrated. Challenges of the HBcAg system due to allostery-induced conformational changes at the intermolecular binding locations were discussed and addressed through biased simulations and empirical data.

Future research might apply this generic framework to other systems of macromolecular self-assembly, especially those difficult to study experimentally because of short timescales or probabilistic structural organization, such as in multi-enzymatic complexes. Additionally, interface phenomena (e.g., adsorption of proteins at oil-water interfaces) and fluid flow can readily be integrated in the framework reported here, e.g., through coupling to computational fluid dynamics. Further improvements might focus on the underlying MD models, incorporation of gradient information in the potential estimate, or improved sampling methods during structural formation and MD binding (e.g., replica exchange). Additionally, finer coarse-graining approaches in MD might incorporate the same general concept of shifting complexity from many 1D distance-based interactions to a single gradient operation on a more complex data-driven potential field. In this regard, the golden mean between granularity of the model and computational resources should always be considered.

## Figures and Tables

**Table 1:** Binding locations between HBcAg${}_{2}$ from the reference capsid (see Figure 1). Positions with respect to the body frame of the reference (molecule A) on *x*-, *y*-, *z*-axis are in nanometer, while angles α,β,γ are in radian.

#	*x*	*y*	*z*	α	β	γ
1	−2.74	−0.74	−3.10	−0.48	0.98	−0.32
2	1.47	−0.91	−4.14	−0.88	−1.05	0.67
3	−3.01	−0.70	−3.08	−2.72	−1.05	3.03
4	−0.65	−0.77	4.25	2.72	0.92	2.76

**Table 2:** Anisotropic translational ($D_{t}$) and rotational ($D_{r}$) diffusion coefficients for HBcAg${}_{2}$ at 293 K and 150 mM NaCl used for MDEM (marked in light orange in Figure 3).

$D_{t}$ [μm${}^{2}$ s${}^{-1}$]			$D_{r}$ [Mrad${}^{2}$ s${}^{-1}$]		
***x***	***y***	***z***	α	β	γ
87.69	72.27	71.48	12.05	7.46	7.00

## Data Availability

The data presented in this study are available on request from the corresponding author. The data are not publicly available due to size (approx. 10 TB).

## Supplementary Materials

The supporting information can be downloaded at https://www.mdpi.com/article/10.3390/ijms232314699/s1. Videos of structural self-assembly are provided for all concentrations at https://doi.org/10.15480/336.4676.

## Abbreviations

The following abbreviations are used in this manuscript:

AA-MD	All-Atom Molecular Dynamics
ANN	Artificial Neural Networks
BD	Brownian Dynamics
BLUE	Best Linear Unbiased Estimate
CG-MD	Coarse-Grained Molecular Dynamics
CPU	Central Processing Unit
DEM	Discrete Element Method
DOF	Degree of Freedom
DPD	Dissipative Particle Dynamics
FF	Force Field
GPR	Gaussian Process Regression
GPU	Graphics Processing Unit
HBcAg	Hepatitis B Core Antigen
HBcAg${}_{2}$	HBcAg Dimer
HBV	Hepatitis B Virus
LD	Langevin Dynamics
MDEM	Molecular Discrete Element Method
MD	Molecular Dynamics
NPT	Isothermal-Isobaric Ensemble
PBC	Periodic Boundary Conditions
PDB	Protein Data Bank
PME	Particle Mesh Ewald
PW	Polarizable Water
QM/MM	Quantum Mechanics/Molecular Mechanics
RMS	Root-Mean-Square
RMSD	Root-Mean-Square Distance
SAS	Self-Assembled Structure
SI	Supplementary Information
SPX	Simulation Procedure X
SVD	Singular Value Decomposition
UK	Universal Kriging
VLP	Virus-Like Particles
