# Supplementary material for: Hierarchical Coarse-Grained Strategy for Macromolecular Self-Assembly: Application to Hepatitis B Virus-Like Particles
Source: Int J Mol Sci. 2022 Nov 24;23(23):14699. doi: 10.3390/ijms232314699 (PMC9740473; doi:10.3390/ijms232314699)
Supplement: Supplementary file 1 [file ijms-23-14699-s001.zip › ijms-2038874-supplementary.pdf]

Supplementary Information

-

Hierarchical Coarse-Grained Strategy for Macromolecular Self-Assembly: Application to Hepatitis B Virus-Like-Particles

Philipp Nicolas Depta[1,*], Maksym Dosta[1,2], Wolfgang Wenzel[3], Mariana Kozlowska[3], Stefan Heinrich[1]

[1]*Institute of Solids Process Engineering and Particle Technology (SPE), 21073 Hamburg University of Technology, Hamburg, Germany*

[2]*Boehringer Ingelheim Pharma GmbH & Co Kg., 88400 Biberach an der Riss, Germany*

[3]*Institute of Nanotechnology (INT), Karlsruhe Institute of Technology,*

76344 Eggenstein-Leopoldshafen, Germany

[*]E-mail: nicolas.depta@tuhh.de

S1 Self-Assembly Supplementary

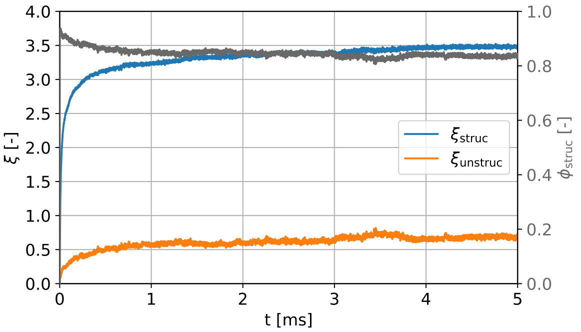

(a) 1 µm^3 with 5 µM.

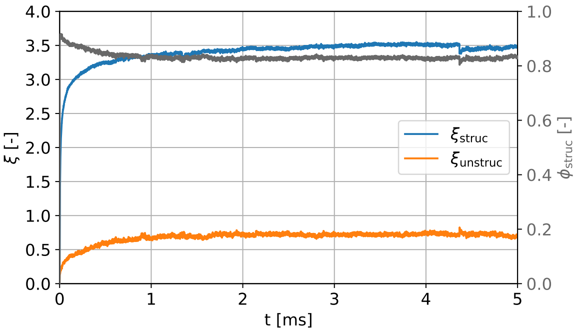

(b) 1 µm^3 with 10 µM.

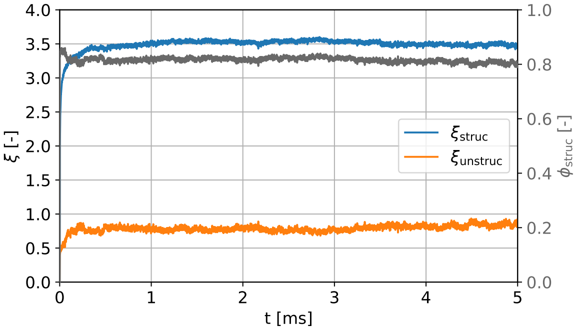

(c) 0.125 µm^3 with 50 µM.

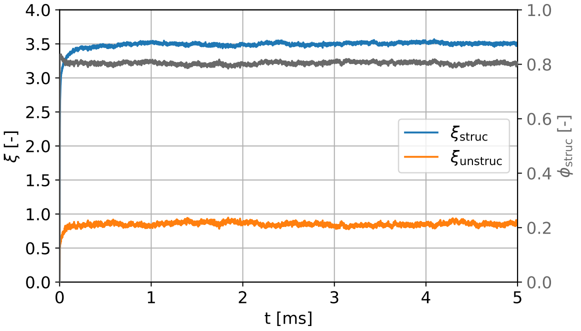

(d) 0.125 µm^3 with 100 µM.

Figure S1: Average structured and unstructured contacts per dimer (left axis) and relative to each other (right axis). Perfect 120-mer without any thermal fluctuations features $\xi_{struc} = 4$.

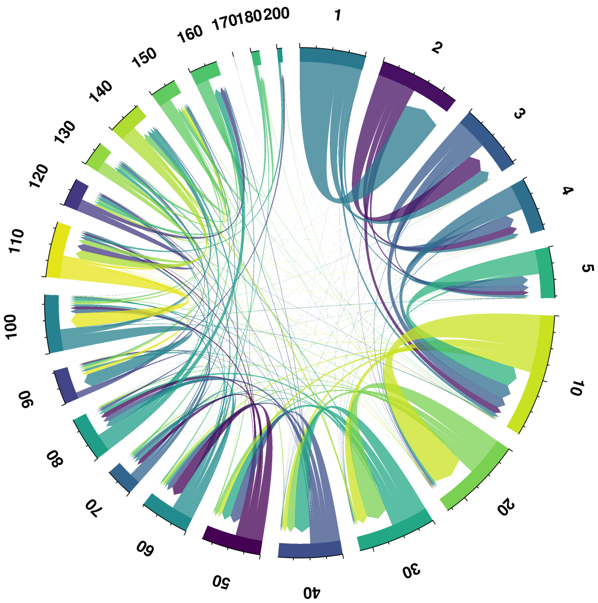

(a) 1 µm^3 with 5 µM.

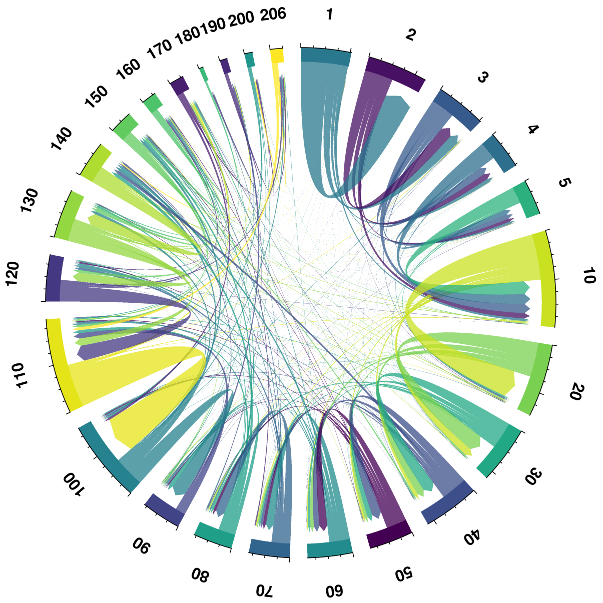

(b) 1 µm^3 with 10 µM.

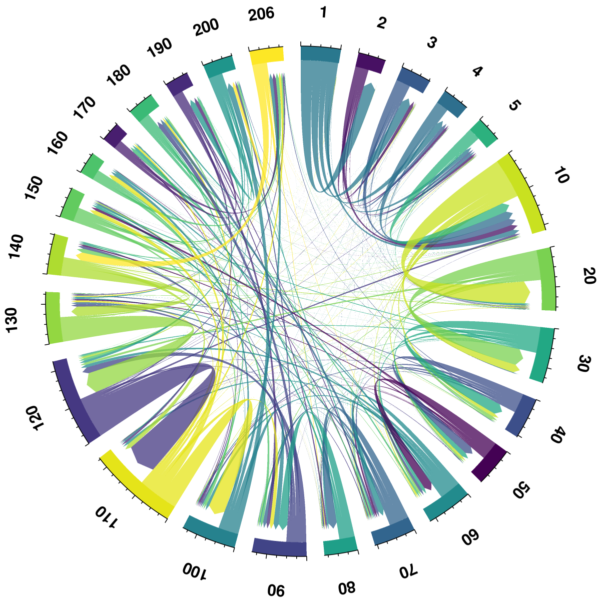

(c) 0.125 µm^3 with 50 µM.

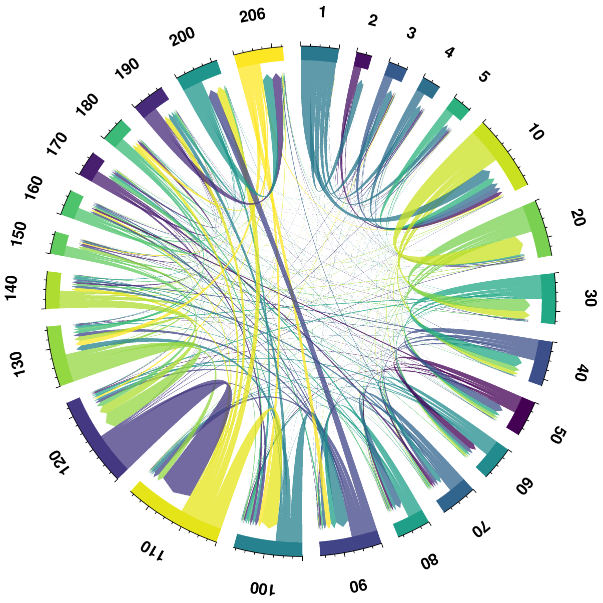

(d) 0.125 µm^3 with 100 µM.

Figure S2: Self-assembly by <u>net transitions</u> between size classes normalized by number of dimers (major ticks represent unit arrow thickness, i.e. every dimer makes this transition on average). Starting at class 10, the size denotes the class range between -4 to +5 relative to the noted value; 206 incorporates all sizes equal to or larger than 206. Colors provide contrast only.

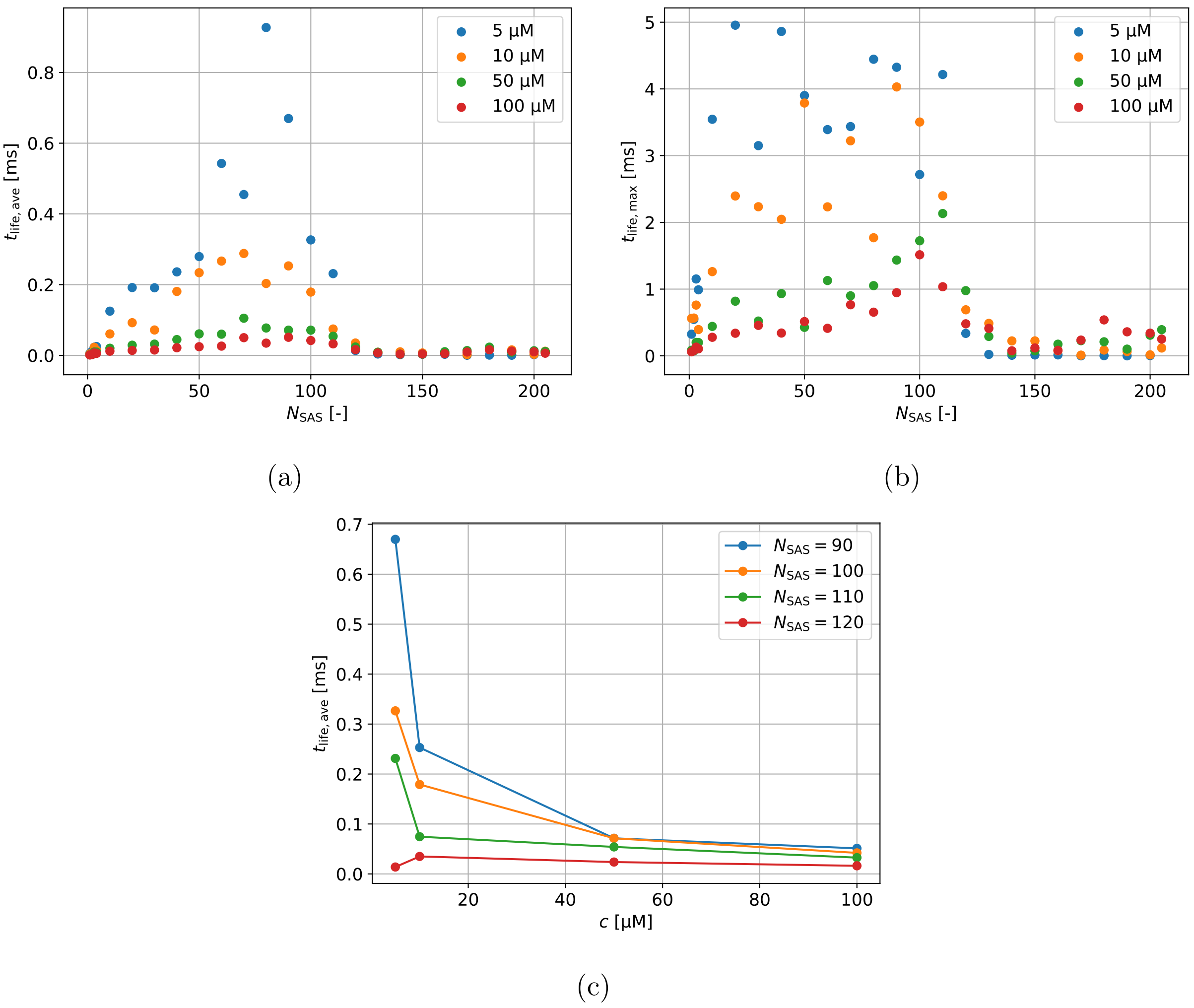

Figure S3: Average and maximum lifetimes of structures.

S2 Methods Supplementary

S2.1 Spatial Descriptors

A - B: In the context of molecule interaction and trend modeling, main focus lies on describing binding events which occur at small distances between molecules. Consequently, capturing the distance between molecules is most crucial. Additionally, the proximity intensity is important, i.e. how extensive / specific the contact and binding site between two molecules is. Furthermore, a spatial descriptor of A-B has to be able to capture the general case where A and B are not equal. With this motivation in mind, three A-B descriptors were investigated:

- δ_m : the minimum distance between the back-bone carbon atoms of A and B subtracted by the collision distance $d_{\text{coll,bb}} = 0.305$ nm;
- $\overline{\delta_m}$: the average δ_m of all back-bone carbon atoms of B to A;
- $\overline{\delta}$: the average distance between all A and B back-bone carbon atoms.

δ_m has the advantage of capturing distance well and starting at zero, while having the disadvantage of not capturing proximity intensity well. In contrast, $\overline{\delta_m}$ and $\overline{\delta}$ have the advantage of capturing proximity intensity well, while having the disadvantage of starting at a non-zero value determined by the molecular structure. This can lead to complex trends with multiple modes, as well as posing issues for colliding conformations. Overall, δ_m was chosen as it provides a simple descriptor over all ranges including collisions.

B - B: A spatial descriptor between two configurations of B is especially important to have a one dimensional distance measure between MD samples, i.e. the spatial similarity / distance between two MD simulations. As A is always at the origin (reference frame), a distance measure is needed to describe the combined translational and rotational distance between two locations of B. As the structure is the same (both B), the well established root-mean-squared-deviation (RMSD) of the N_B back-bone atoms of B is used:

$$\delta_r = \sqrt{\frac{1}{N_B}\sum_{i=1}^{N_B}\delta^2_{i,B1-B2}} \tag{S1}$$

S2.2 Basic Functions for Trend and Variogram Fitting

The following basic functions are used in the context of this work. For trend modeling, a continuous, non-singular, and asymptotic function is required, which is fulfilled by the

constant, exponential, spherical, Gaussian, cubic, and generalized logistic function (GLF). For variogram modeling, additional requirements exist (e.g. conditional definiteness). Therefore, the typically used basic functions in literature were used, i.e. exponential, spherical, Gaussian, and cubic. Furthermore, MD box size compensation was performed using a basic linear function. In accordance with the nomenclature typically used in literature n is termed nugget, s sill, r range, and h the heaviside function.

$$\nu = x/r, \qquad \chi = x - r \ , \tag{S2}$$
$$f_{\text{const}}(x) = c \ , \tag{S3}$$
$$f_{\text{lin}}(x) = a + bx \ , \tag{S4}$$
$$f_{\text{exp}}(x) = n + (s-n)(1 - e^{-3\nu}) \ , \tag{S5}$$
$$f_{\text{sph}}(x) = n + (s-n)(h(-\chi) \times (1.5\nu - 0.5\nu^3) + h(\chi)) \ , \tag{S6}$$
$$f_{\text{gauss}}(x) = n + (s-n)(1 - e^{-x^2 r^{-2/3}}) \ , \tag{S7}$$
$$f_{\text{cubic}}(x) = n + (s-n)(h(-\chi) \times (7\nu^2 - 8.75\nu^3 + 3.5\nu^5 - 0.75\nu^7) + h(\chi)) \ , \tag{S8}$$
$$f_{\text{GLF}}(x) = n + (s-n)(1/(1 + e^{-B\chi})) \tag{S9}$$

A variogram fit is considered invalid if $n > s$, $r > 5$ nm, $\sigma(r) > 0.2r$, $\sigma(s) > 0.2|n-s|$, or $\sigma(n) > 0.2|n-s|$. A trend fit is considered invalid if $r_{95} > 4$ nm, $r_{95} < 0.2$ nm, $\sigma(r) > 0.2r$, $\sigma(s) > 0.2|n-s|$, $\sigma(n) > 0.2|n-s|$, or for GLF $\sigma(B) > 0.25B$, where r_{95} is the range when $f = n + 0.95(s-n)$ for GLF.

S2.3 Kriging Size Convergence

Table S1: Convergence study of the number of Kriging points N_{krig} in reference to $N_{krig} = 1000$ using the $HBcAg_2$ random data set and grid for iterative refinement (within cutoff and outside of collisions).

Parameter	100	250	500	1000
Mean potential dif. [kJ/mol]	-0.064	0.042	0.065	0
Min potential dif. [kJ/mol]	-80.5	-33.0	-19.0	0
Max potential dif. [kJ/mol]	62.3	46.3	21.4	0
Mean variance dif. [kJ^2/mol^2]	5.9	2.5	0.8	0
Min variance dif. [kJ^2/mol^2]	-0.32	-0.19	-0.002	0
Max variance dif. [kJ^2/mol^2]	309.5	103.0	31.6	0

S2.4 Grid Design

In the context of grid design, three aspects are important: the structure of both molecules, the interaction cutoff distance, and memory constraints for saving the grid (specifically on GPUs). Based on these three aspects, an optimal homogeneous overall grid design can be determined. The Cartesian extend of the overall box can be estimated by the maximum extend of molecule A in x, y, and z (positive and negative) increased in all directions by the cutoff distance of interaction and maximum distance of any atom of B from its COM (i.e. extend over all orientations). Furthermore, the orientation equivalents have to be determined, which are resulting from the molecular structure of B. For this, the maximum distance of each axis in combination with the respective circular section have been used, leading to the strictest orientation resolutions. The orientation space itself is 2π for α and γ, and π for β. Based on this, the homogeneous overall grid encompassing the full interaction space can be derived, which is fully described by a single grid resolution. E.g. a 0.2 nm grid refers to such a homogeneous overall grid.

S2.5 Molecular Collisions and Objective Function for Structural Stability

The main concept of the model for molecular collisions is that the increase in interaction potential caused by overlapping molecules is correlated with the number of collisions of back-bone atoms $N_{\mathrm{coll,bb}}$ (distance below $d_{\mathrm{coll,bb}} = 0.305$ nm) and to some extend of side-chain atoms $N_{\mathrm{coll,non-bb}} = N_{\mathrm{coll,full}} - N_{\mathrm{coll,bb}}$ ($N_{\mathrm{coll,full}}$ is number of all atom collisions resulting from distances below $d_{\mathrm{coll,full}} = 0.4$ nm). The collision distances are motivated by the force-field and equilibrium distances of carbon atoms in the back-bone structure. In addition, molecular flexibility to avoid collisions has to be accounted. For this, the distance of a configuration to the next MD data point d_{MD} can be used. Hence, the increase in interaction potential resulting from molecular collisions is modeled by super-position on the potential derived by

Universal Kriging U_K as

$$\begin{aligned}
U &= U_\mathrm{K} + l(d_\mathrm{MD}) \times \\
&\quad (c_\mathrm{rep,bb} \times h(\kappa_\mathrm{bb}) \times \kappa_\mathrm{bb} + c_\mathrm{rep,non-bb} \times h(\kappa_\mathrm{non-bb}) \times \kappa_\mathrm{non-bb}) \ , && \text{(S10)} \\
\kappa_\mathrm{bb} &= G(N_\mathrm{coll,bb}, w_\mathrm{coll}) - N_\mathrm{min,bb} \ , && \text{(S11)} \\
\kappa_\mathrm{non-bb} &= G(N_\mathrm{coll,non-bb}, w_\mathrm{coll}) - N_\mathrm{min,non-bb} \ , && \text{(S12)} \\
l(d_\mathrm{MD}) &= \begin{cases} 1 & \text{if } d_\mathrm{MD} > d_\mathrm{MD,min} + w_\mathrm{MD,min} \\ 0 & \text{if } d_\mathrm{MD} < d_\mathrm{MD,min} \\ \frac{d_\mathrm{MD} - d_\mathrm{MD,min}}{w_\mathrm{MD,min}} & \text{else} \end{cases} && \text{(S13)}
\end{aligned}$$

where $l(d_\mathrm{MD})$ is a linear scaling function depending on the distance to the next MD data point d_MD between $d_\mathrm{MD,min}$ and $d_\mathrm{MD,min} + w_\mathrm{MD,min}$, h is the heaviside function, G is a Gaussian smoothing kernel with a width w_coll, $N_\mathrm{min,bb}$ and $N_\mathrm{min,non-bb}$ are the minimum number of back-bone and non-back-bone collisions, and lastly $c_\mathrm{rep,bb}$ and $c_\mathrm{rep,non-bb}$ are the repulsion coefficients for back-bone and non-back-bone collisions. Parameters of the repulsion model were then derived by a combination of optimization and parameter studies using an objective function of the HBcAg capsid stability. The objective function O_stab was derived by combining multiple structural parameters in reference to the starting structure of a capsid (Δ indicating deviation) averaged over the last ten time steps as

$$\begin{aligned}
O_\mathrm{stab} = \langle 2|\Delta r_\mathrm{gyr}| + \frac{|\Delta d_\mathrm{com,min}|}{3} + \frac{|\Delta d_\mathrm{com,max}|}{3} \\
+ 2|\Delta d_\mathrm{i,minDistAnyJ,rmsd}| + |\Delta d_\mathrm{ij,min}| + |\Delta d_\mathrm{ij,rmsd}|\rangle_{10}, \quad \text{(S14)}
\end{aligned}$$

where r_gyr is the radius of gyration, $d_\mathrm{com,min}$ / $d_\mathrm{com,max}$ are the minimum / maximum distance between the COM of the capsid and any molecule COM, $d_\mathrm{i,minDistAnyJ,rmsd}$ is the root-mean-square of the minimum distance of all molecules i to any j, $d_\mathrm{ij,min}$ is the minimum distance between any i and j permutations, and $d_\mathrm{ij,rmsd}$ is the root-mean-square-distance of all i and j permutations. Therefore, the objective function captures structural extend as well as internal conformation. O_stab has a value of zero for perfect agreement of the structure with the reference structure and increases with increasing structural differences.

The best parameters for accounting of molecular collisions were found to be $c_\mathrm{rep,bb} = 50$ kJ/mol, $N_\mathrm{min,bb} = 0.25$, $d_\mathrm{MD,min} = 0.4$ nm, $w_\mathrm{MD,min} = 0.5$ nm, $w_\mathrm{coll} = 0.3$ nm, which were consistently used for all interaction potentials.

S2.5.1 Initial Sampling and Iterative Refinement

Initial Sampling Initial sampling was performed to generate a data set sufficient for statistical analysis including variogram estimation, i.e. spatial correlation. Due to the complex structure of the interaction hyper-space interesting within the 6D interaction space, straight forward application of literature methods such as latin hypercube sampling is not possible. Furthermore, variogram estimation requires at least a number of data points to be in proximity to each other in order to estimation spatial correlation at small distances. Most (semi-)random sampling strategies are opposed to this. Additionally, the interaction space expands with increasing distance between molecules, while small distances are most crucial for interaction. In order accommodate these ideas during initial sampling, a systematic random sampling approach at varying distances classes was implemented. For this, the two molecules were placed with random positions and orientations inside a 100 nm box until the minimum distance between any of their atoms d_{A-B} matched a prescribed interval. The number of samples for each class can be found in Tab. S2. This approach allowed for example to put a partial emphasis on binding locations (0.4 - 0.5 nm) or the main binding region (0.5 - 2.5 nm).

Table S2: Initial random sampling over distances classes for $HBcAg_2$ - $HBcAg_2$ data set. Note that due to the symmetry of interaction (A = B), the number of samples is essentially double (or interaction space half in volume).

d_{A-B} [nm]		# samples
lower	upper	
0.4	0.5	20’000
0.5	0.7	5’000
0.7	0.9	5’000
0.9	1.1	5’000
1.1	1.3	5’000
1.3	1.5	5’000
1.5	1.7	5’000
1.7	1.9	5’000
1.9	2.1	5’000
2.1	2.3	5’000
2.3	2.5	5’000
2.5	3.0	5’000
3.0	3.5	5’000
3.5	4.0	5’000
4.0	4.5	5’000
4.5	5.0	5’000

Furthermore, a proximity resampling method was developed, which repeats MD data points multiple times, resulting in slightly different end configurations. These runs are especially useful to improve variogram estimation at small relative distances. However, in the context of $HBcAg_2$ sampling density was sufficient to not require this.

Iterative Refinement In order to iteratively refine the potential field in a near-optimal fashion, the field knowledge and qualities of Universal Kriging are exploited - most notably the estimation variance and possibility to calculate the impact of an additional data point on the variance without knowledge of the data value. Overall, refinement was performed based on five criteria for a total of 29 iterations, leading to 375'000 MD data points:

- **Iteration 1 - 10: Variance minimization.** In the first step, ten iterations of 5'000 samples each are performed to reduce the field variance and avoid false negatives, i.e. missing e.g. a binding location. For this, the field location outside of the collision region with the largest variance is searched, a virtual data point placed at it (new resampling point), the variance in the affected region recalculated using Universal Kriging, and the next variance maxima searched. This is repeated until the desired (5'000) resampling points are found. Although this approach is strictly speaking not mathematically optimal, it provides a sufficiently good and computationally reasonable algorithm to reduce the field variance.

- **Iteration 11 - 20: Normalized variance minimization.** In the second step, ten iterations of 5'000 samples each are performed to reduce the normalized field variance (normalized within each variogram region). The same algorithm as for the variance is used and main advantage lies in also improving the field estimate at large distances between molecules, which inherently posses a reduced variance.

- **Iteration 21, 24, 27: Potential minima localization and quantification.** In the third step, three iterations of 20'000 samples each are performed to localize and quantify potential minima, i.e. binding locations. For this, an extrema search is conduced including the identification of all grid points leading to this extrema, called the neighborhood or attractive region. 15'000 resampling points are then placed at the main extrema and 5'000 at random first-level neighborhood points (neighboring grid points) of the extrema according to the size of their neighborhood (maximum limited to 25 %).

- **Iteration 22, 25, 28: Potential maxima localization and quantification.** In the fourth step, three iterations of 20'000 samples each are performed to localize and

quantify potential maxima, i.e. repulsive locations. The same procedure as for potential minima is carried out.

- **Iteration 23, 26, 29: Gradient maxima localization and quantification.** In the fifth step, three iterations of 20'000 samples each are performed to localize and quantify gradient maxima, i.e. attractive/repulsive locations, defined by the maximum absolute difference to a neighboring grid point. The same procedure as for potential minima is carried out.

Consequently, refinement was performed to improve the overall estimate and avoid false negatives, as well as to properly locate and quantify regions of interest, such as binding locations (potential minima) and repulsive regions (potential maxima and gradient maxima).

S2.5.2 Force and Torque Numerical Gradient Operation

The translational gradient (force) can be calculated in second-order approximation using the central differences as

$$-\nabla_{\mathrm{t},i} U = -\frac{U(\vec{x} + \hat{i}h_i, \vec{\theta}) - U(\vec{x} - \hat{i}h_i, \vec{\theta})}{2h_i}, \tag{S15}$$

where $i \in \{x, y, z\}$ indicates the direction, $\hat{i}$ the unit vector in each direction, and h_i the grid resolution in i. Note that for readability the index '$body, A \leftarrow B$' was dropped. Higher-order gradients were tested but did not show noticeable differences for the investigated grid resolutions in final results, which is attributed to the noise introduced by the diffusion model. When calculating the gradient, knowledge of the potential at arbitrary locations is necessary, which does not necessary have to coincide with the grid. Two approaches were investigated in this regard: The first approach performed linear interpolation in 6D to estimate the potential at intermediate locations and required significantly more computing cycles and memory bandwidth, leading to almost a one order increase in computation time depending on platform. The second approach used a nearest-neighbor approximation, which significantly reduces computing cycles and memory bandwidth, but creates discrete jumps in the gradient for coarse grids. While energy conservation of the first approach was found to be superior in the absence of the diffusion model, no significant differences justifying the computational increase could be found in the presence of the diffusion model.

The rotational gradient (torque) requires a more complex calculation due to the coupling with translation and conversion of a gradient in Eulerian space via the respective Jacobian.

The gradient can be calculated in first-order approximation using central differences as

$$-\overrightarrow{\nabla_{\mathrm{r}} U} = -\begin{bmatrix}\cos(\beta)\cos(\gamma) & -\sin(\gamma) & 0\\ \cos(\beta)\sin(\gamma) & \cos(\gamma) & 0\\ -\sin(\beta) & 0 & 1\end{bmatrix}\begin{bmatrix}\nabla_{\mathrm{r},\alpha} U\\ \nabla_{\mathrm{r},\beta} U\\ \nabla_{\mathrm{r},\gamma} U\end{bmatrix}, \tag{S16}$$

$$\nabla_{\mathrm{r},i} U = \frac{U(\mathbf{T}^{-1}\vec{x}, \vec{\theta} + \hat{i}\phi_i/2) - U(\mathbf{T}\vec{x}, \vec{\theta} - \hat{i}\phi_i/2)}{2\phi_i}, \tag{S17}$$

where the preceding matrix is the Jacobian at the relative orientation α, β, γ. The orientation step ϕ_i with $i \in \{\alpha, \beta, \gamma\}$ is essentially distributed over A and B and the resulting change in relative position calculated using the relative rotation matrix $\mathbf{T}$ from $\vec{\theta}$ to $\vec{\theta} + \hat{i}\phi_i/2$.

Special attention has to be paid at boundaries of the grid domain. A wrap-around was implemented for periodic degrees of freedom (α and γ) and back-mapping for non-periodic degrees of freedom. After calculation of the potential gradients in the body frame of reference of A, rotation into the global reference frame for overall force balance is required.

S2.5.3 Critical Time Step

The critical time step of the employed methodology was estimated separately for the intermolecular interaction model through eigenfrequency analysis and for the diffusion model by the previously established correlations in Depta et al. [DOI: 10.1021/acs.jcim.8b00613]. For the intermolecular interaction, the oscillation period of the corresponding two-mass spring system was estimated using the second-order partial derivative ${}^2\nabla$ for translation t and rotation r of the potential field U as

$$\tau_{\text{crit,interaction}} = 2\pi \cdot min\left(\sqrt{\frac{m}{max(\|{}^2\overrightarrow{\nabla_{\mathrm{t}} U}\|)}}, \sqrt{\frac{I_{\min}}{max(\|{}^2\overrightarrow{\nabla_{\mathrm{r}} U}\|)}}\right), \tag{S18}$$

where m is the dimer mass and $I_{\min}$ the minimum component of the mass moment of inertia (thus I_α). The second-order partial derivatives were calculated equivalently to the gradient operation for forces and torques using a second-order central finite difference scheme with nearest-neighbor approximation. Note that the chosen approximation represents the worst-case assumption with regard to the rotational component. This conservative approach was found to be sufficient in light of dominating diffusive components over the calculation of reduced mass moments of inertia in the respective second-gradient directions at all grid location. Based on this estimation, the critical time step was found to be 2.9×10^{-13} s for the diffusion model (2.9×10^{-12} s for a dynamic viscosity reduced by $\times 0.1$), 5.3×10^{-12} s for the translational component of the interaction model, and 2.0×10^{-12} s for the rotational

component of the interaction model.

S3 $HBcAg_2$ Interaction Potential

S3.1 Pure MD-based Interaction Potential

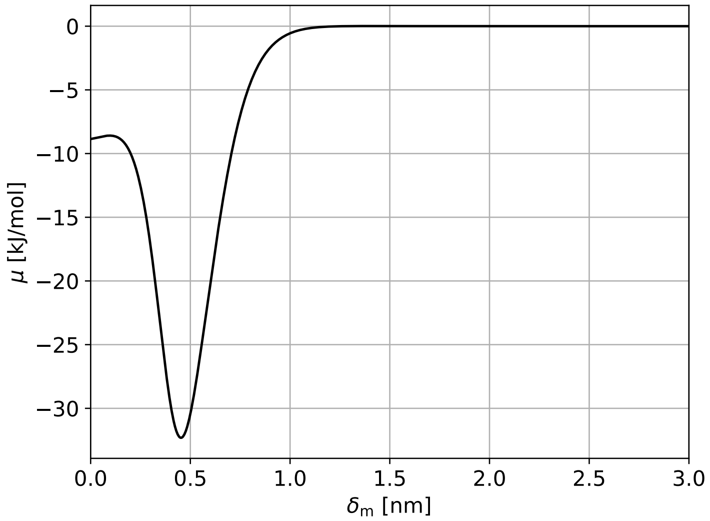

Figure S4: Sum of all minimum distance trends μ, i.e. without detailed residual potential R.

S3.2 Biased MD-based Interaction Potential

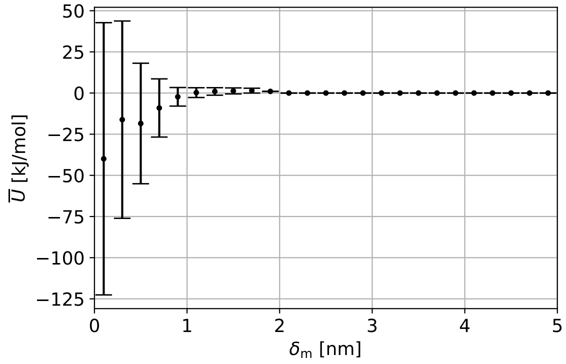

(a) Grid average and standard deviation binned over minimum distance.

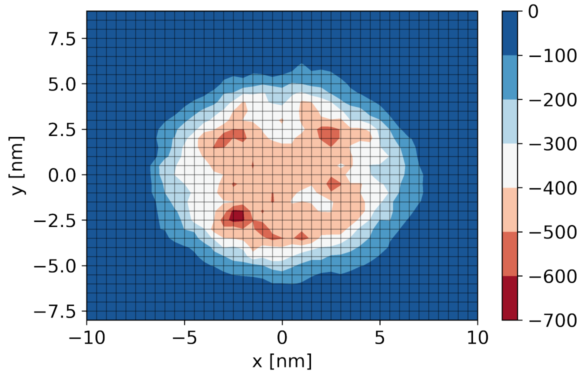

(b) X-Y cross-section minimum over all remaining dimensions.

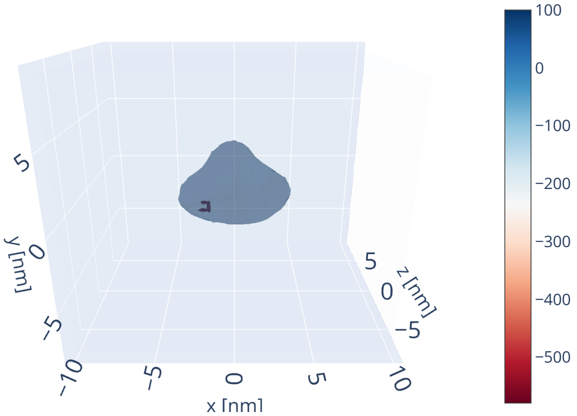

(c) 3-D minimum over orientations.

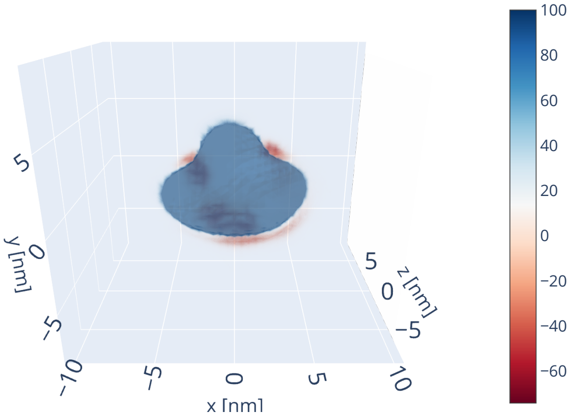

(d) 3-D mean over orientations.

Figure S5: Potential field visualizations based on biased MD sampling at binding locations.

S3.3 Kriging Statistical Data

In the following, the statistical data for the Kriging algorithm is shown including smoothed trends (using a Gaussian kernel of width 0.213 nm derived from the structural variability of the molecule structure captured by the standard deviation of the difference between rigid structural minimum distance and MD minimum distance), trend fits, as well as variogram data and fits for all sections. Failed variogram fits are shown with placeholders. Note that not all shown trend and variogram fits are considered valid for Kriging purposes. See Sec. S2.2 for requirements. Valid trends are A-B, A-PW + B-PW, PW-PW, A-ion + B-ion, PW-ion, Bond. Not valid trends are A-A + B-B, ion-ion, G96-angles, improper dihedral angles, reciprocal coulomb potential. Valid variogram models were only those for potential A-B besides that above range. Noise in trends towards large δ_m is attributed to low sampling density.

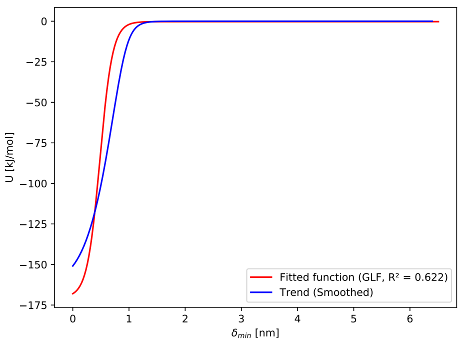

(a) Potential trend.

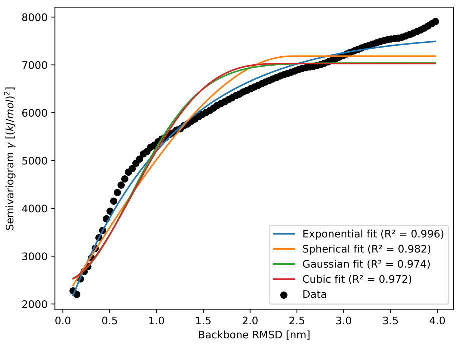

(b) Overall variogram.

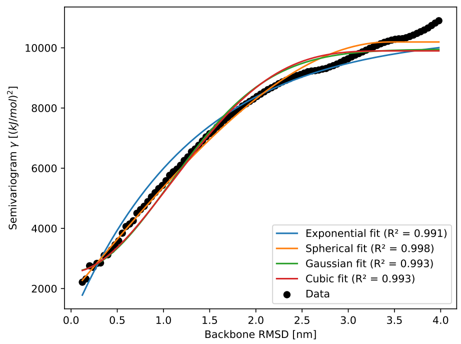

(c) Variogram section 0.

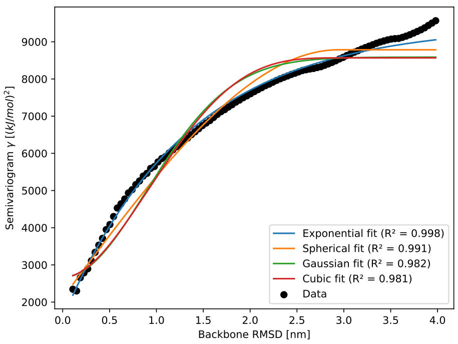

(d) Variogram section 1.

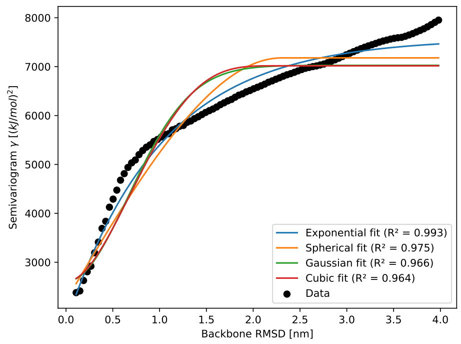

(e) Variogram section 2.

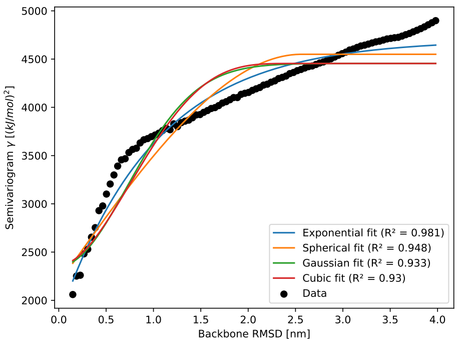

(f) Variogram section 3.

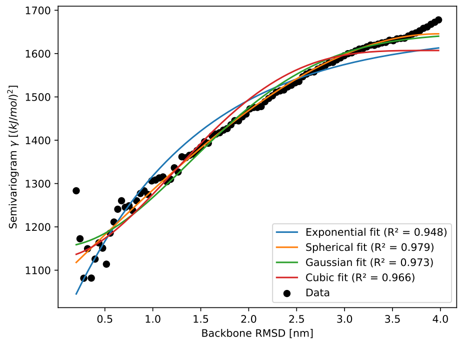

(g) Variogram section 4.

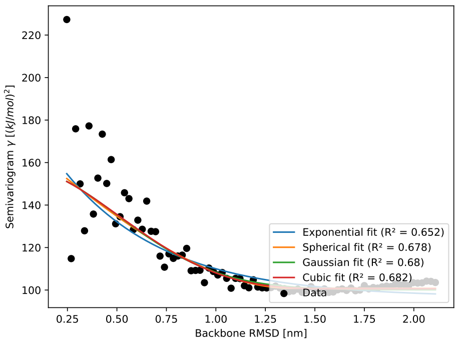

(h) Variogram above range.

Figure S6: Potential A-B.

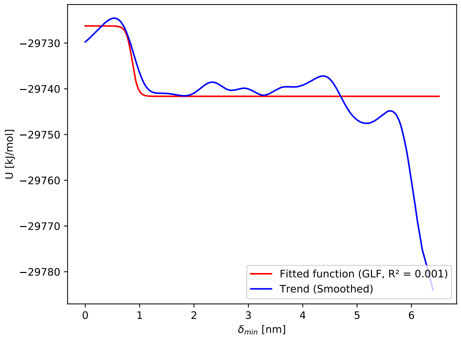

(a) Potential trend.

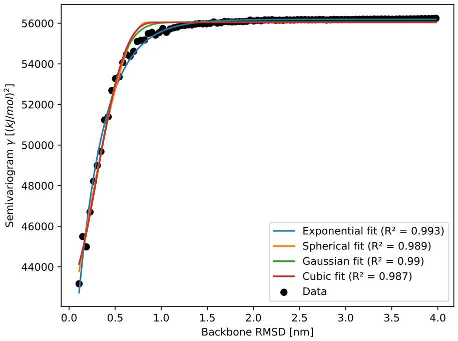

(b) Overall variogram.

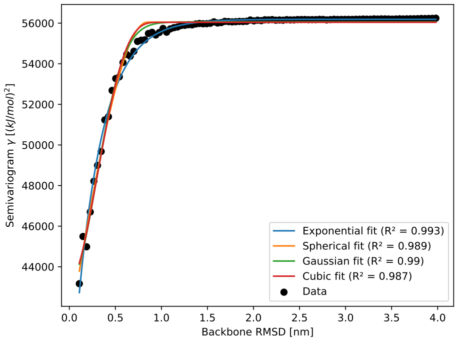

(c) Variogram section 0.

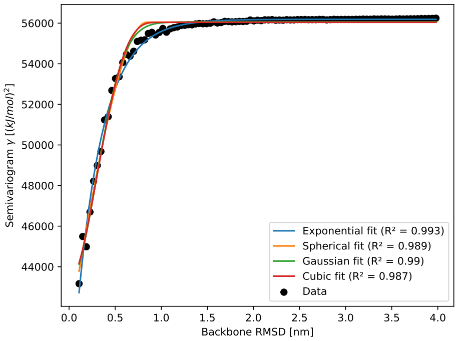

(d) Variogram section 1.

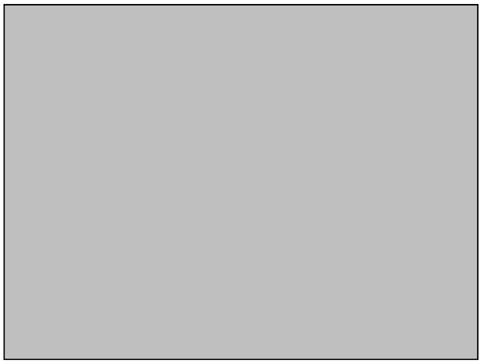

(e) Variogram section 2.

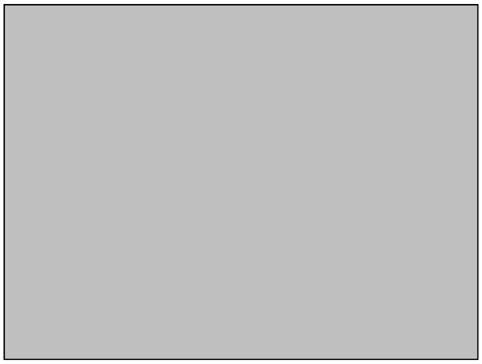

(f) Variogram section 3.

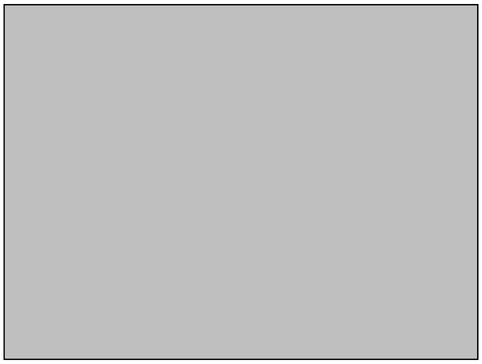

(g) Variogram section 4.

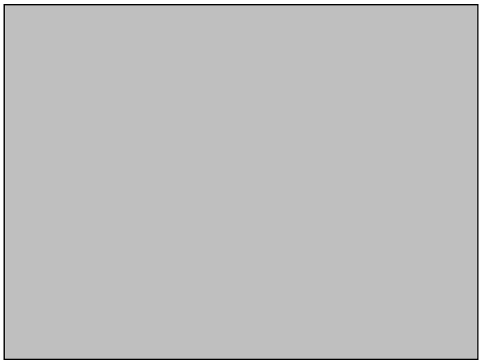

(h) Variogram above range.

Figure S7: Potential A-A + B-B.

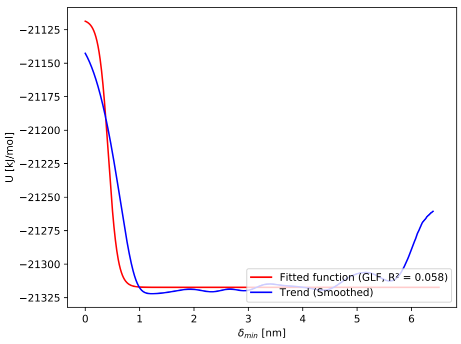

(a) Potential trend.

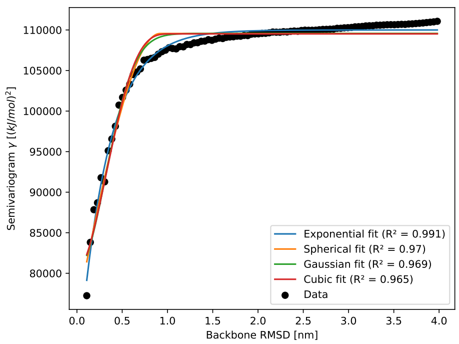

(b) Overall variogram.

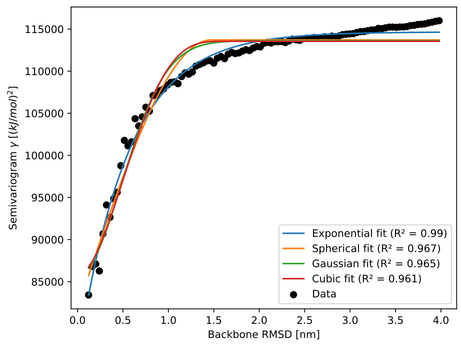

(c) Variogram section 0.

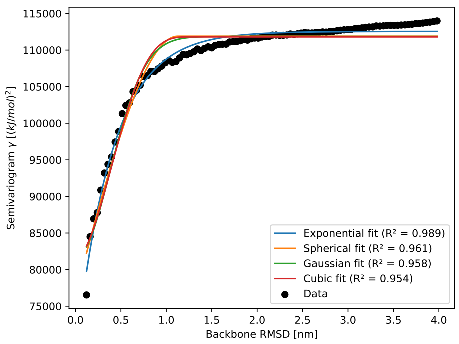

(d) Variogram section 1.

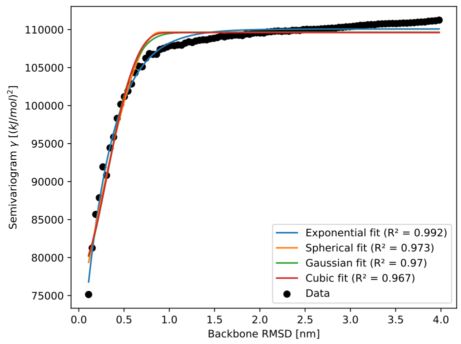

(e) Variogram section 2.

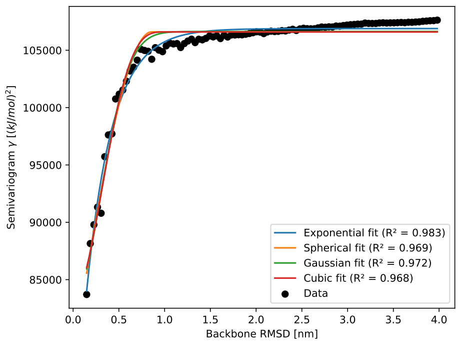

(f) Variogram section 3.

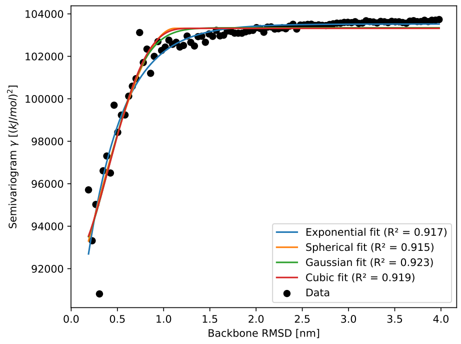

(g) Variogram section 4.

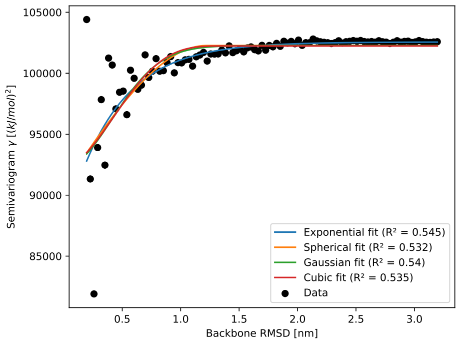

(h) Variogram above range.

Figure S8: Potential A-PW + B-PW.

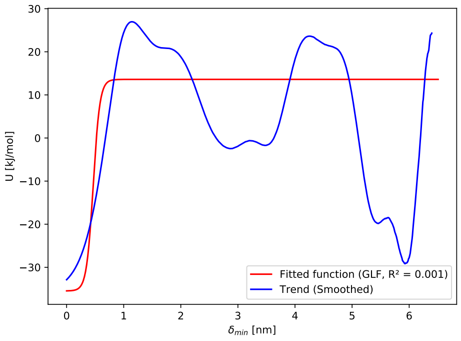

(a) Potential trend.

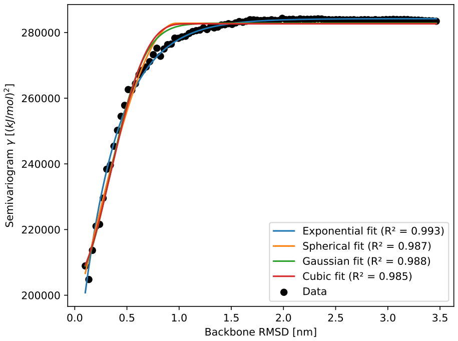

(b) Overall variogram.

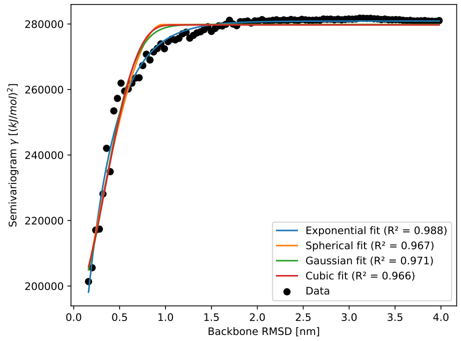

(c) Variogram section 0.

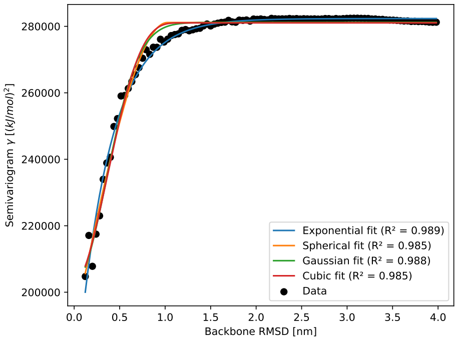

(d) Variogram section 1.

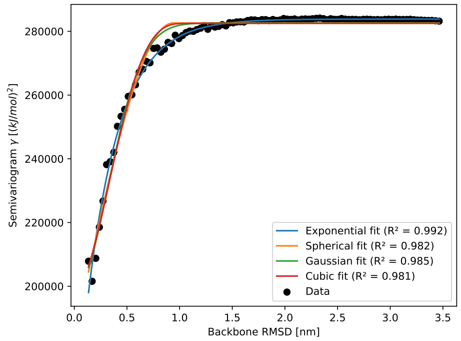

(e) Variogram section 2.

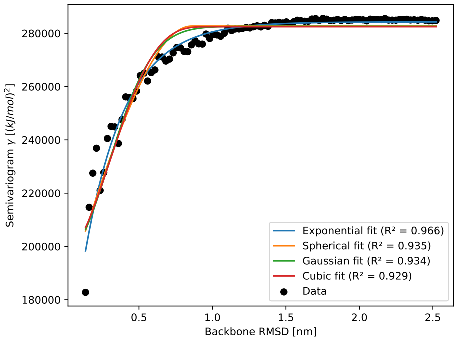

(f) Variogram section 3.

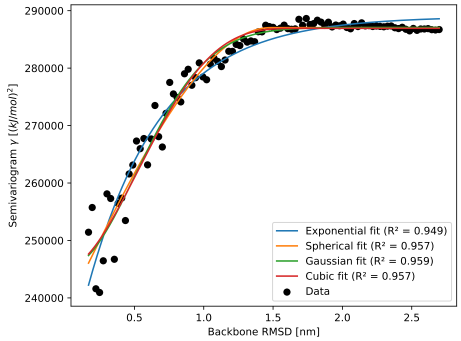

(g) Variogram section 4.

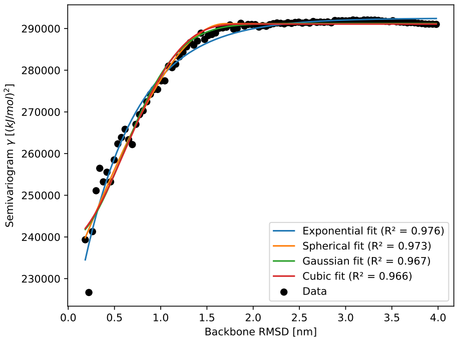

(h) Variogram above range.

Figure S9: Potential PW-PW.

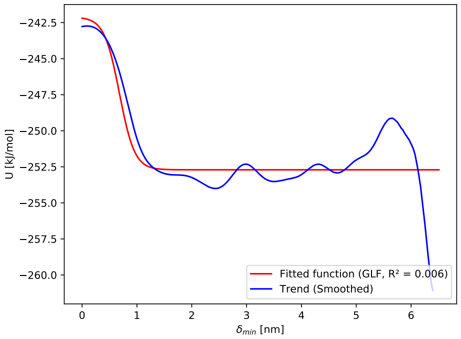

(a) Potential trend.

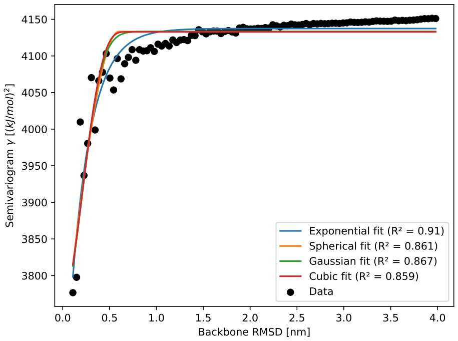

(b) Overall variogram.

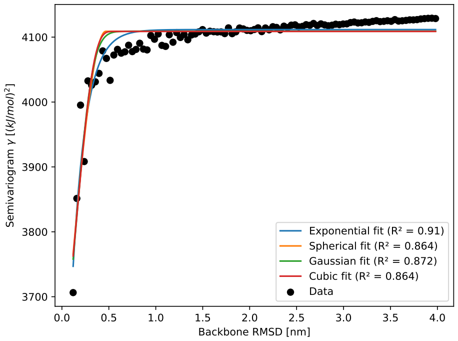

(c) Variogram section 0.

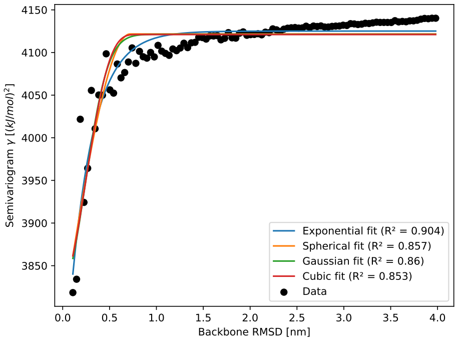

(d) Variogram section 1.

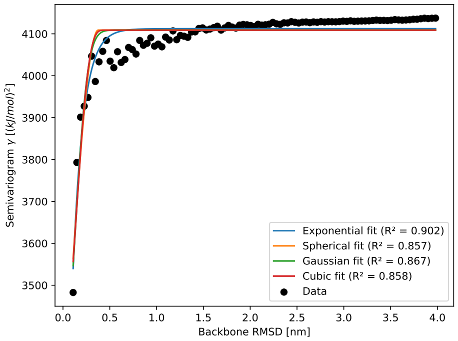

(e) Variogram section 2.

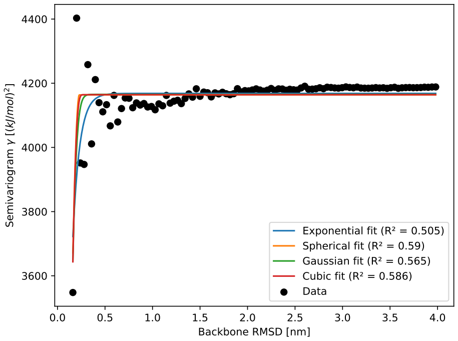

(f) Variogram section 3.

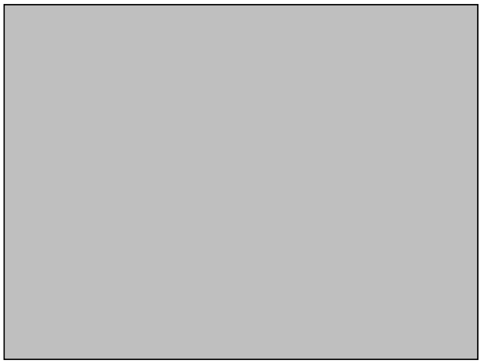

(g) Variogram section 4.

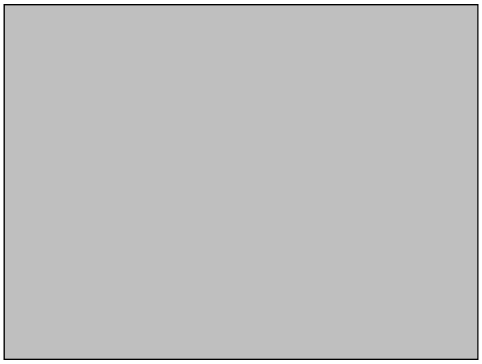

(h) Variogram above range.

Figure S10: Potential A-Ion + B-Ion.

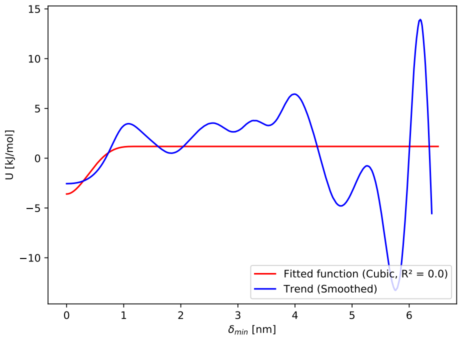

(a) Potential trend.

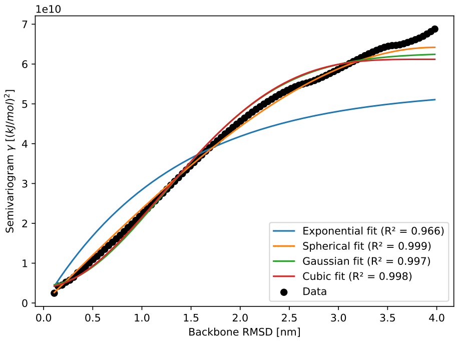

(b) Overall variogram.

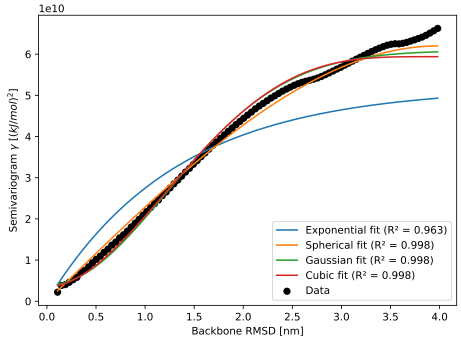

(c) Variogram section 0.

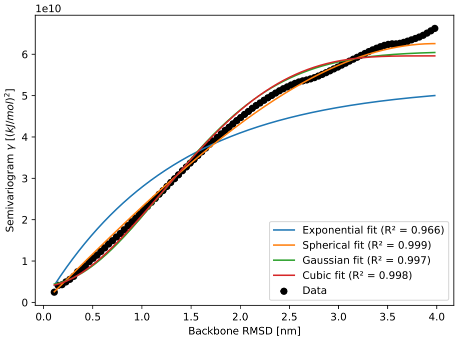

(d) Variogram section 1.

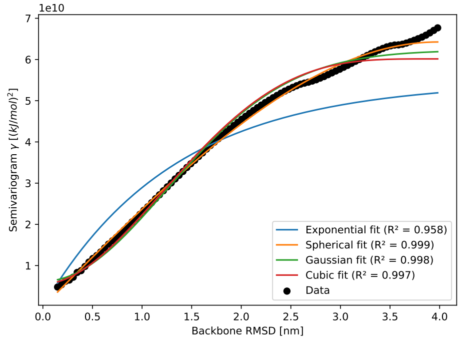

(e) Variogram section 2.

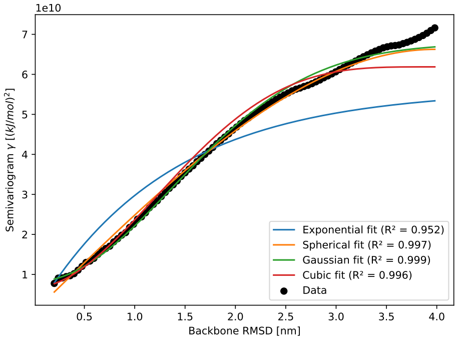

(f) Variogram section 3.

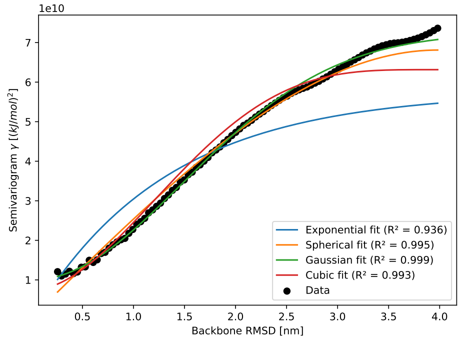

(g) Variogram section 4.

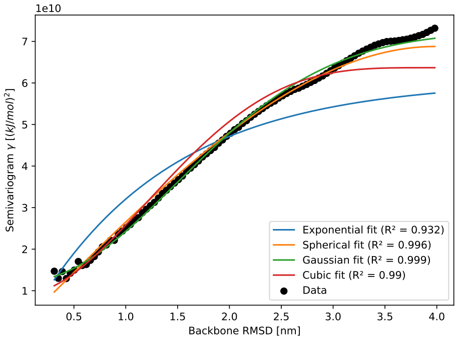

(h) Variogram above range.

Figure S11: Potential PW-Ion.

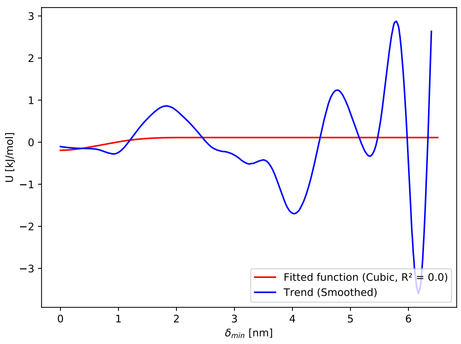

(a) Potential trend.

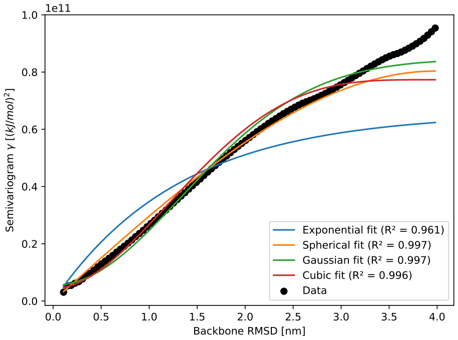

(b) Overall variogram.

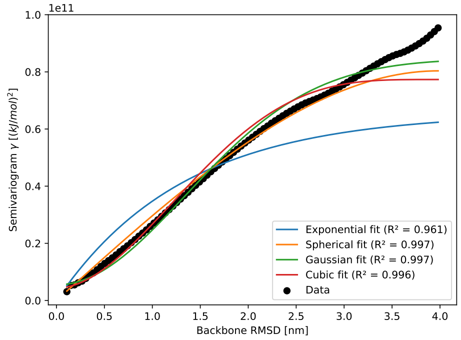

(c) Variogram section 0.

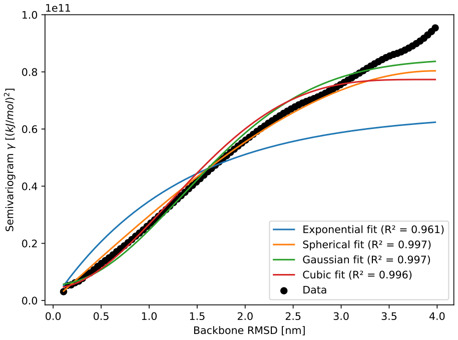

(d) Variogram section 1.

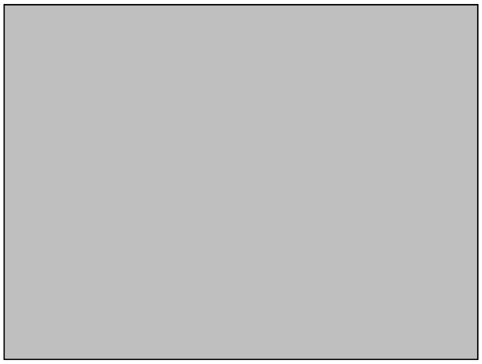

(e) Variogram section 2.

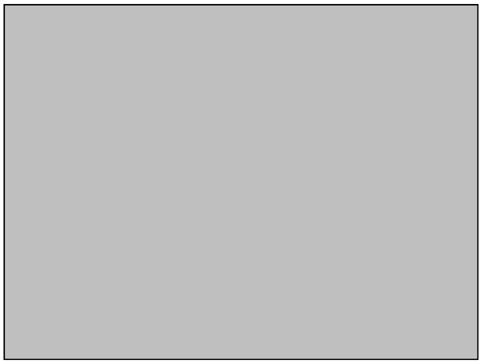

(f) Variogram section 3.

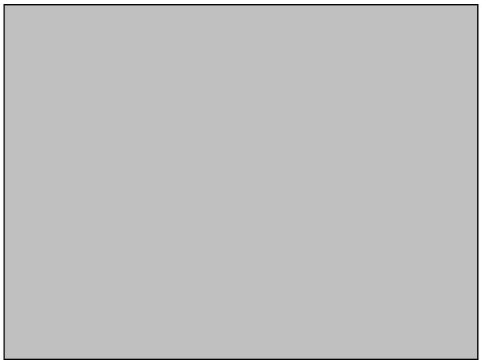

(g) Variogram section 4.

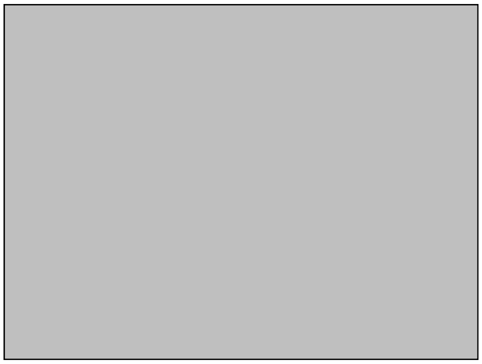

(h) Variogram above range.

Figure S12: Potential Ion-Ion.

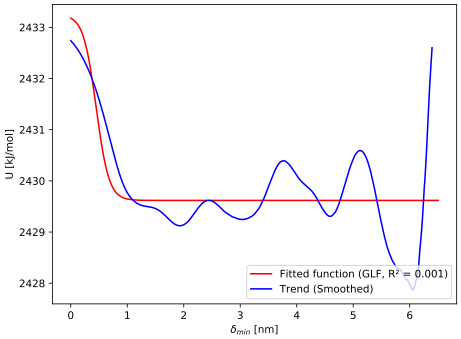

(a) Potential trend.

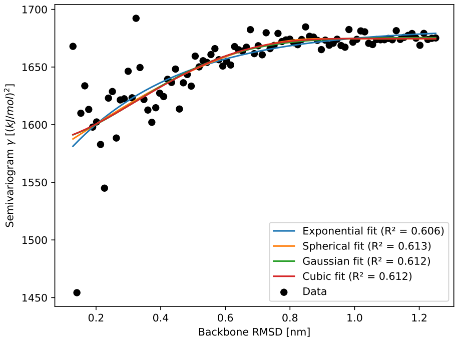

(b) Overall variogram.

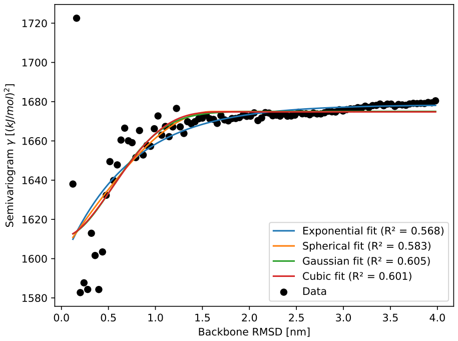

(c) Variogram section 0.

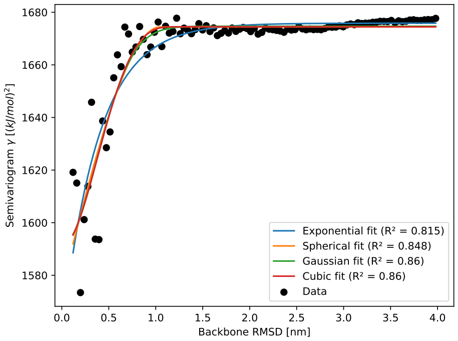

(d) Variogram section 1.

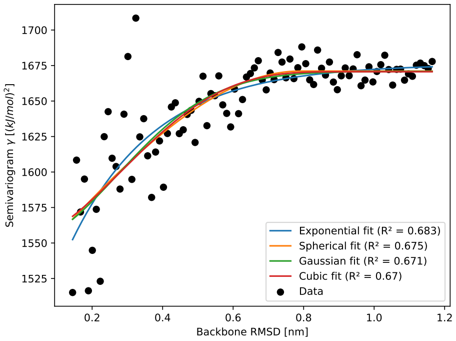

(e) Variogram section 2.

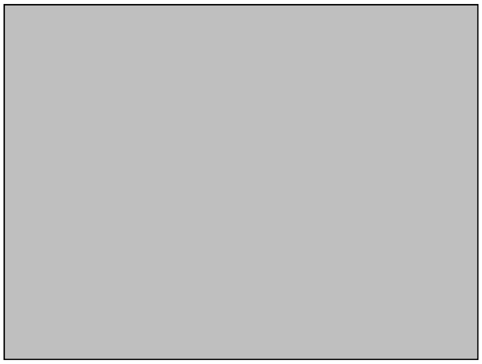

(f) Variogram section 3.

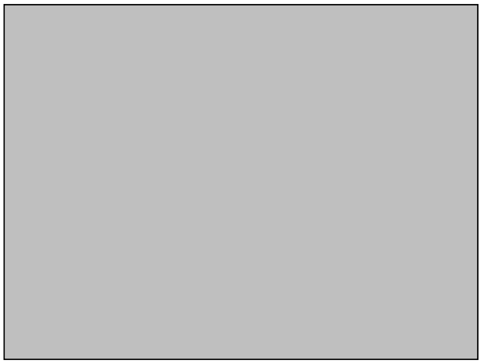

(g) Variogram section 4.

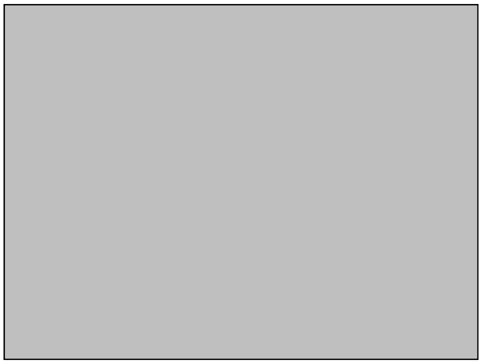

(h) Variogram above range.

Figure S13: Potential Bonds.

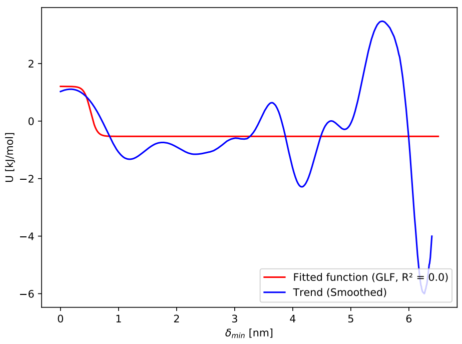

(a) Potential trend.

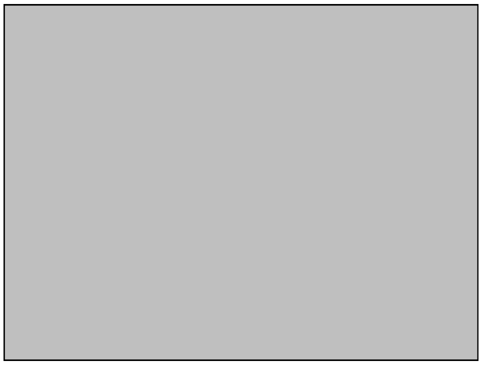

(b) Overall variogram.

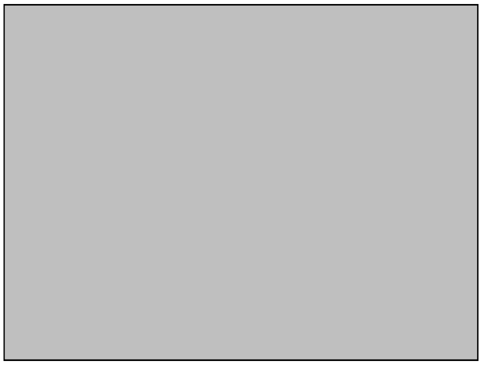

(c) Variogram section 0.

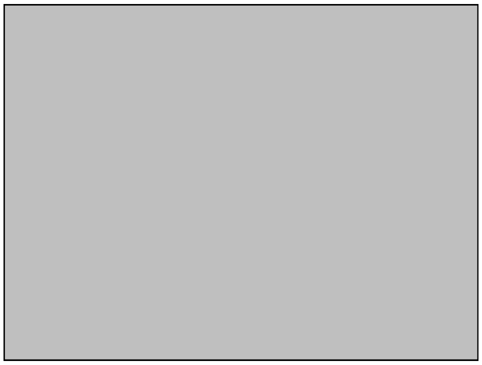

(d) Variogram section 1.

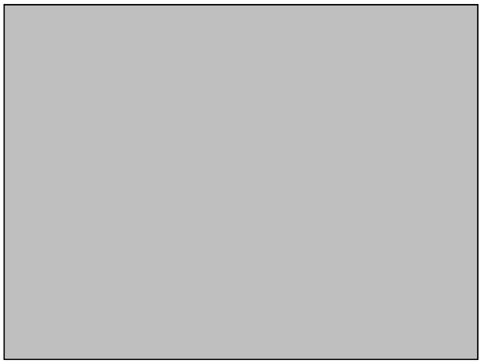

(e) Variogram section 2.

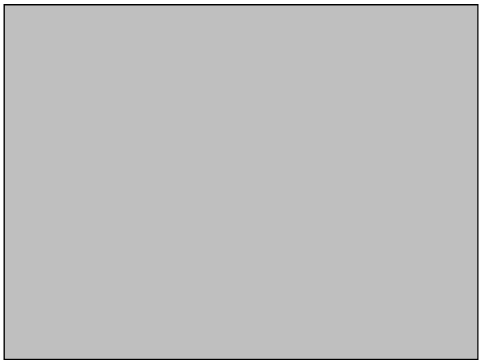

(f) Variogram section 3.

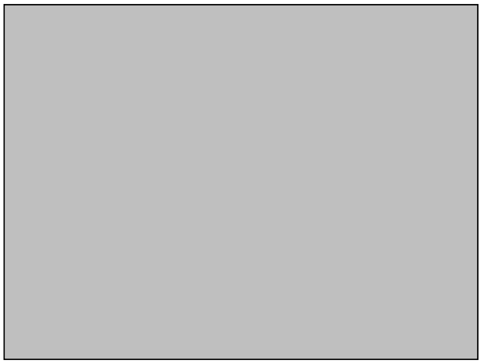

(g) Variogram section 4.

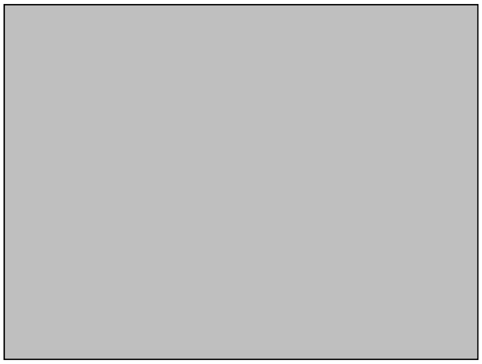

(h) Variogram above range.

Figure S14: Potential G96-Angle.

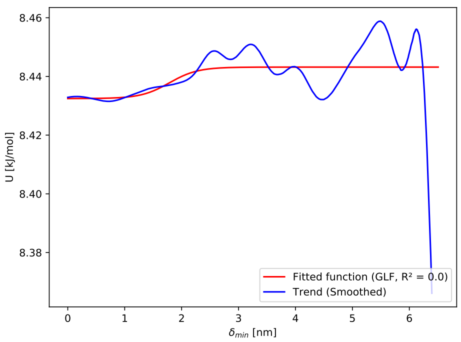

(a) Potential trend.

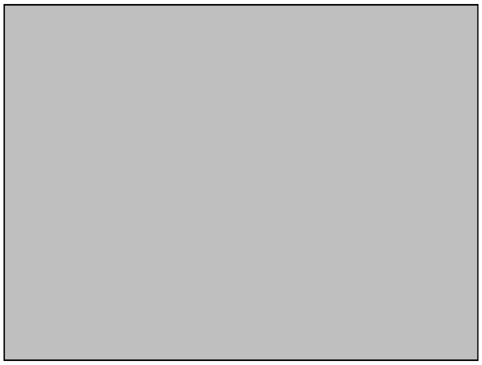

(b) Overall variogram.

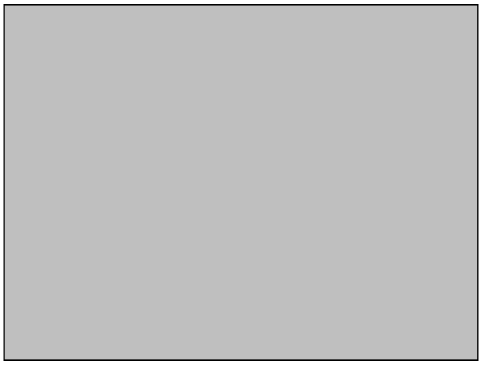

(c) Variogram section 0.

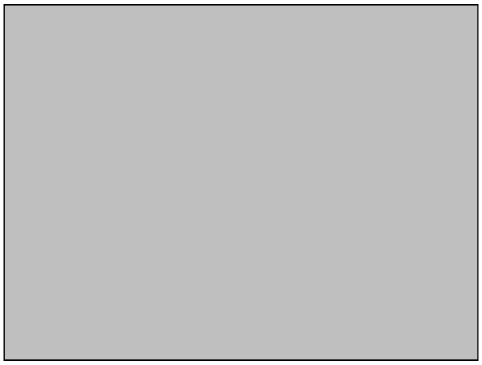

(d) Variogram section 1.

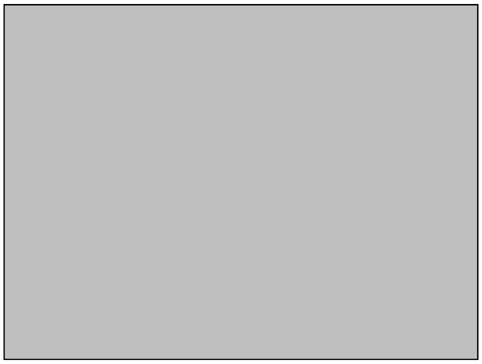

(e) Variogram section 2.

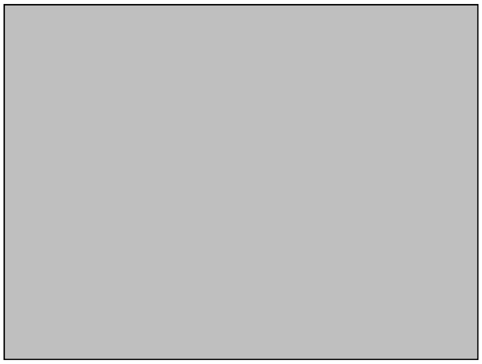

(f) Variogram section 3.

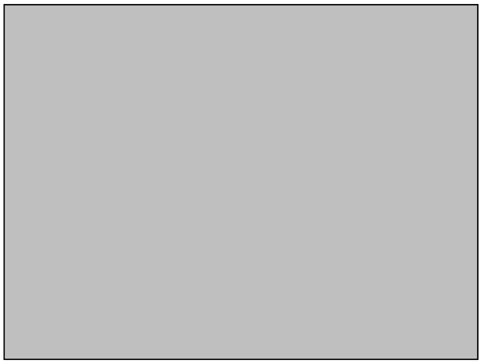

(g) Variogram section 4.

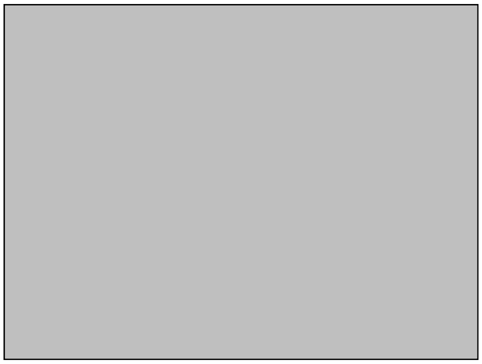

(h) Variogram above range.

Figure S15: Potential improper dihedral angles.

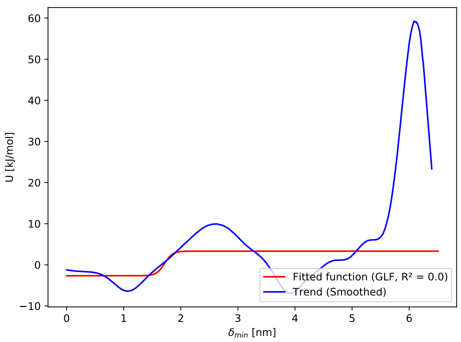

(a) Potential trend.

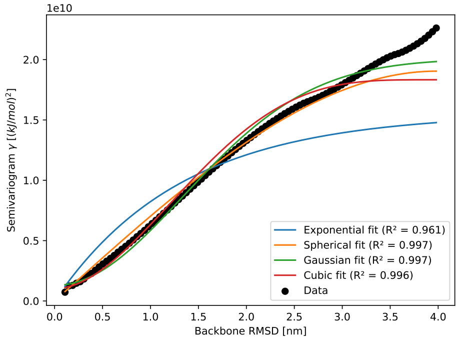

(b) Overall variogram.

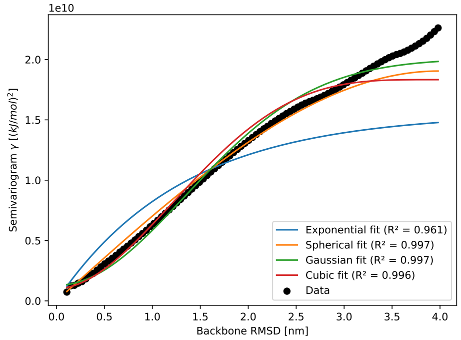

(c) Variogram section 0.

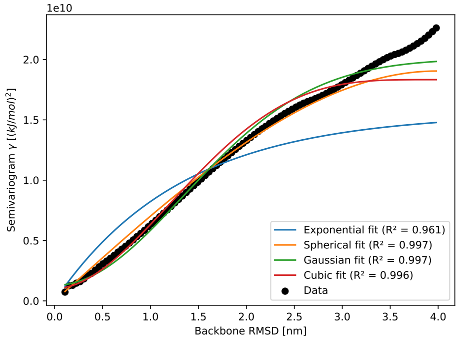

(d) Variogram section 1.

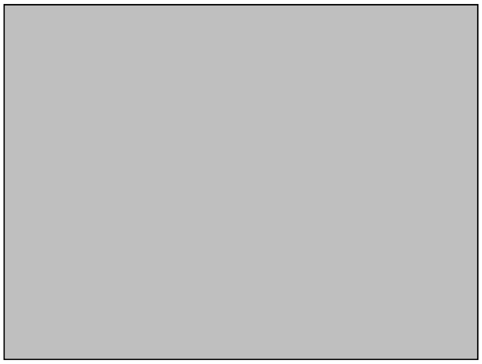

(e) Variogram section 2.

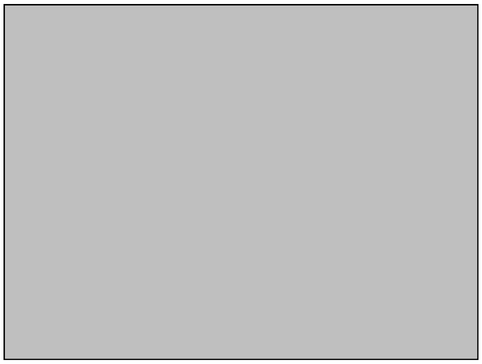

(f) Variogram section 3.

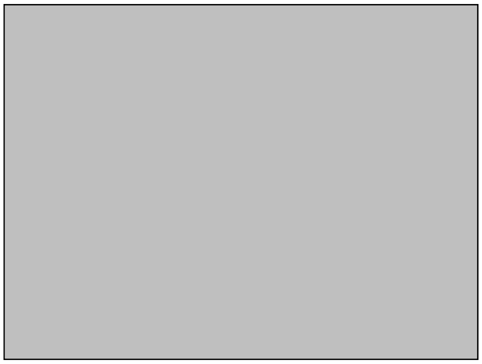

(g) Variogram section 4.

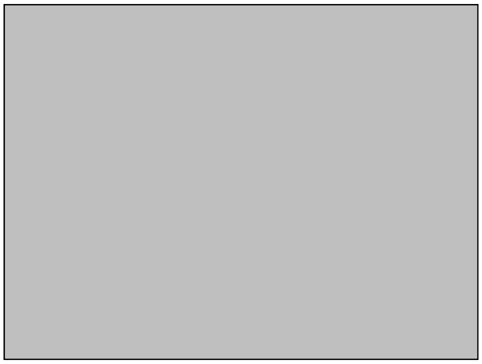

(h) Variogram above range.

Figure S16: Potential Coulomb reciprocal.
